# Naive Prediction of Protein Backbone Phi and Psi Dihedral Angles Using Deep Learning

**DOI:** 10.3390/molecules28207046

**Published:** 2023-10-12

**Authors:** Matic Broz, Marko Jukič, Urban Bren

**Affiliations:** 1 Faculty of Chemistry and Chemical Engineering, University of Maribor, Smetanova ulica 17, SI-2000 Maribor, Slovenia; 2 Faculty of Mathematics, Natural Sciences and Information Technologies, University of Primorska, Glagoljaška ulica 8, SI-6000 Koper, Slovenia; 3 Institute of Environmental Protection and Sensors, Beloruska ulica 7, SI-2000 Maribor, Slovenia

**Keywords:** protein structure prediction, backbone dihedral angles, deep neural network, fully connected neural network (FCNN), ϕ and ψ angle prediction, protein secondary structure prediction

## Abstract

Protein structure prediction represents a significant challenge in the field of bioinformatics, with the prediction of protein structures using backbone dihedral angles recently achieving significant progress due to the rise of deep neural network research. However, there is a trend in protein structure prediction research to employ increasingly complex neural networks and contributions from multiple models. This study, on the other hand, explores how a single model transparently behaves using sequence data only and what can be expected from the predicted angles. To this end, the current paper presents data acquisition, deep learning model definition, and training toward the final protein backbone angle prediction. The method applies a simple fully connected neural network (FCNN) model that takes only the primary structure of the protein with a sliding window of size 21 as input to predict protein backbone ϕ and ψ dihedral angles. Despite its simplicity, the model shows surprising accuracy for the ϕ angle prediction and somewhat lower accuracy for the ψ angle prediction. Moreover, this study demonstrates that protein secondary structure prediction is also possible with simple neural networks that take in only the protein amino-acid residue sequence, but more complex models are required for higher accuracies.

## 1. Introduction

**Figure 1:**
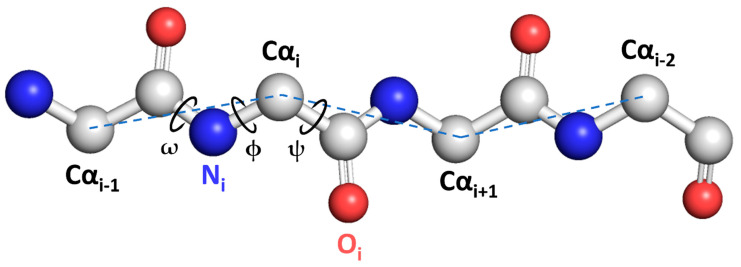
A schematic representation of ϕ, ψ, and ω protein backbone dihedral angles. Grey spheres represent carbon atoms, blue spheres nitrogen atoms, and red spheres oxygen atoms.

Proteins are commonly composed of 20 natural amino acid residues, which together form the primary protein structure. Each amino-acid residue contains the common atoms N, Cα, and C that comprise the protein backbone, or main chain. As illustrated in Figure 1, the backbone structure of amino-acid residues can be described by sets of ϕ (phi), ψ (psi), and ω (omega) dihedral angles. These angles are defined by considering four consecutive backbone atoms from the sequence C_i−1_, Ni, Cα_i_, C_i_, N_i+1_, and C_αi+1_. In order to simplify models, the ω angle can be typically fixed at 180° [1]. Each amino-acid residue has a side chain emanating from its Cα atom; however, secondary structure prediction studies typically disregard it and rather focus on the protein backbone.

Predicting the three-dimensional structure of a given protein from its sequence, known as protein structure prediction (PSP), has presented a major challenge in biochemistry for decades. In 2020, DeepMind released AlphaFold, which has become the most accurate algorithm to date for tackling this problem [2]. AlphaFold’s exceptional performance in the free modeling (FM) section of the thirteenth critical assessment of protein structure prediction (CASP) ignited heightened public interest [3,4]. At CASP14, the most up-to-date variant of AlphaFold entered under the group name “AlphaFold2”, showed tremendous progress in accuracy, thereby setting a new benchmark for sub-Ångström root mean square deviation (Cα r.m.s.d.) backbone predictions [5,6]. Before AlphaFold revolutionized PSP, numerous algorithms for accurately predicting protein secondary structure were developed and remain indispensable, both for template-free and template-based protein structure predictions [6,7,8,9,10]. 

Protein secondary structure prediction (PSSP) is commonly viewed as a categorization problem wherein each amino acid residue is classified according to its secondary structure type. PSSP models accept a sequence of amino-acid residues as input and return the corresponding sequence of secondary structures. They can be classified into several types depending on the number of secondary structure categories, with three-state (Q3) and eight-state (Q8) models being the most common. In the three-state PSSP model, the secondary structure elements are composed of two main conformations, helix (H) and sheet (E) [11], plus the coil (C) category representing the amino-acid residues that fall into neither of the previous two categories. The eight-state PSSP framework, on the other hand, incorporates eight categories of protein secondary structures: α-helix (H), 3_10_-helix (G), parallel/anti-parallel β-sheet conformation (E), isolated β-bridge (B), bend (S), turn (T), π-helix (I), and coil (C) categories, as initially proposed by Kabsch and Sander [12]. Another approach for the classification of protein structures is DISICL [13], a dihedral-based segment identification and classification method that offers 18 distinct structural classes, which can be simplified into seven more general classes, providing a detailed analysis of subtle structural changes. Over the years, numerous methods and algorithms have been developed that reached 70.2–87.3% Q3 accuracies (PHD [14], PSIPRED [15], SPINE [16,17], SPARROW [18], Porter 4.0 [19], SCORPION [20], SPIDER2 [21], Jpred4 [22,23], DeepCNF [24], SPIDER3 [25], MUFOLD-SS [26], NetSurfP-2.0 [27], CRRNN and eCRRNN [28], OPUS-TASS [29], and DNSS2 [30]), but lower Q8 accuracies ranging from 62.6% to 76.5% (SSpro8 [31], RaptorX-SS8 [32], SCORPION [33], ICML2014 [34], DeepCNF [24], MUFOLD-SS [26], CRRNN and eCRRNN [28]), owing to the increasing complexity of the problem.

Despite the recent improvements in predicting ϕ and ψ backbone angles, the obtained ranges of indicative angles are still relatively wide (approximately 20°), which poses a significant challenge when attempting to capture the protein backbone structure accurately. Various methods have been proposed to predict backbone angles as continuous or discrete labels, with the aim of achieving increased accuracy compared to secondary structure prediction for application in ab initio structure prediction or refinement [35,36]. Recent advances in protein backbone angle predictions (BAP) have been made through the use of deep neural networks (DNNs). DNN variants, such as stacked sparse auto-encoder neural networks [37], long short-term memory (LSTM), bidirectional recurrent neural networks (BRNNs) [25,29,38], residual networks (ResNets) [38], and DNN ensembles [29,38] or layered iterations [21], have been utilized for BAP. Common input features for BAP include position-specific scoring matrices (PSSMs) [21,37,38,39,40,41] generated by PSI-BLAST, 7 physicochemical properties (7PCP) [21,37,38,40], such as hydrophobicity and volume, predicted accessible surface area (ASA) [37,41], hidden Markov model (HMM) profiles [27,38,40] (by HHBlits [42]), contact maps [38], and PSP19 [29]. Additionally, to capture local structures around amino-acid residues, many methods use sliding windows [21,25,37,41], while others apply entire protein sequences as features [21,43] to capture long-range interactions. Convolutional neural networks (CNNs) [27,29] or LSTM-BRNNs [25,38] have also been utilized for this purpose.

However, despite these advancements, more accurate BAP is still needed due to the cascading effect of errors at any angle of a protein structure. Consequently, other methods for BAP have been developed, such as ANGLOR [41], SPIDER [37], SPIDER2 [21], SPIDER3 [25], SPOT—Contact [44], RaptorX-Angle [45], DeepRIN [19], NetSurfP-2.0 [27], SPOT-1D [38], OPUS-TASS [29], and SAP [46]. For example, ANGLOR utilizes neural networks and support vector machines (SVMs) [31] to predict ϕ and ψ angles separately, while SPIDER applies a stacked sparse autoencoder DNN for predicting θ (planar angle defined by the consecutive Cα atoms) and τ (dihedral angle defined by four consecutive Cα atoms) angles. RaptorX-Angle uses a combination of clustering and deep learning to predict ϕ and ψ values, and DeepRIN utilizes a deep residual inception network for the same purpose. NetSurfP-2.0, on the other hand, employs large LSTM networks in BRNNs to predict ϕ and ψ angles. Moreover, SPOT-1D applies an ensemble of LSTM-BRNN and ResNets with input features PSSM, HMM, 7PCP, and contact maps; the contact maps are taken from SPOT-Contact [44] and are used in a sliding window fashion. Entire proteins are also applied as features for SPOT-1D. OPUS-TASS predicts only ϕ and ψ angles with ensembles of DNNs having CNN, LSTM, and Transformer [32] layers. It utilizes an input feature called PSP19 [33] that classifies residues into rigid-body blocks and a constrained feature called CSF3 [34] to describe backbone structures. OPUS-TASS also employs a multi-task learning strategy [35]. SAP predicts all four types of backbone angles using a simple fully connected neural network (FCNN) with sliding windows, 8-state SS predictions, PSSM, and 7PCP input features. On the benchmark datasets, SAP4SS [47] has achieved mean absolute error (MAE) values of 15.59°, 18.87°, 6.03°, and 21.71°, respectively, for ϕ, ψ, θ, and τ predictions, which is a slight improvement from SAP [46], which has achieved values of 15.65°, 18.59°, 6.07°, and 21.03°, respectively. As a result, SAP4SS has somewhat outperformed the existing state-of-the-art methods such as SAP, SPOT-1D, and OPUS-TASS, with differences in MAE ranging from 1.5 to 4.1% compared to the best-known results.

The current study applies a simple fully connected neural network (FCNN) that takes only a given primary structure of the protein with a sliding window of size 21 as input features to predict ϕ and ψ angles. Despite its simplicity, the model predicts ϕ dihedral angles with a surprising accuracy of 23.53° MAE but performs somewhat worse for the prediction of ψ dihedral angles (MAE of 44.14°). The study shows that even a naive approach to a simple model can perform with surprising accuracy and can serve to study the primary sequence of proteins while maintaining the transparency of the model and a good overview of the input data. This study also serves to demonstrate how a simple model based on sequence input data could integrate into complex ensemble solutions like AlphaFold.

## 2. Results

Upon finishing model training, the ϕ and ψ angle predictions were made for the test dataset. For each prediction, the loss function formula was applied so that the error calculation accounted for the angle periodicity, and all predicted angles were adjusted either by adding or subtracting 360° so that they fell into the same −180° to 180° range as the measured angles. The results were analyzed using custom Python functions using the Pandas 1.5.1. package [48] for the dataset handling and MatPlotLib 3.6.3 [49] for the graphs.

### 2.1. Mean Absolute Prediction Error

**Figure 2:**
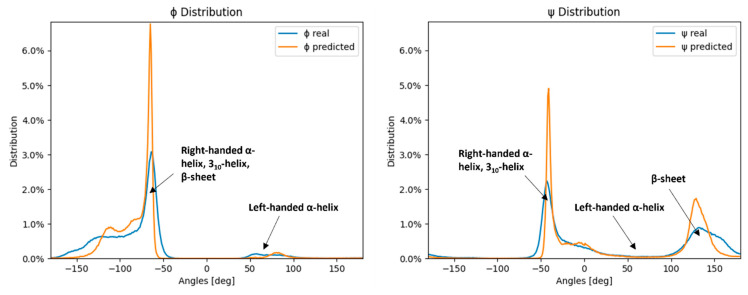
Distribution of measured angles of the testing dataset and neural network predictions. The blue lines represent the measured ϕ and ψ angle distributions of the test dataset, while the orange lines represent the neural network-predicted ϕ and ψ angle distributions of the test dataset.

Figure 2 shows that the distribution of the neural network model predicted values corresponds to the distribution of the measured values for ψ and ϕ angles, with peaks and troughs in both distributions aligning across the range. However, the predicted distributions’ peaks are larger and narrower than the measured distribution peaks, while the predicted distributions’ troughs are wider than the ones of the measured distributions, effectively resulting in a smoothed lower-resolution distribution curve. The neural network model therefore produces similar angle distributions as the ones obtained by the SAP4SS beforehand [47] (depicted in Figure 2).

In Figure 2, where angle distributions are depicted, the peaks represent well-defined secondary structures, such as helices and sheets. The ϕ distribution is heavily skewed toward negative dihedral angle values because the three common secondary structure elements overlap there—right-handed α-helix, 3_10_-helix, and β-sheet—while the small peak around 40° to 100° corresponds to the left-handed α-helix. Conversely, the ψ angle distributions are more evenly divided into two peaks, corresponding to the two most prominent secondary structure elements—right-handed α-helix and β-sheet. Herein, the reason behind less accurate predictions of ψ angles by neural networks can be clearly visualized.

**Figure 3:**
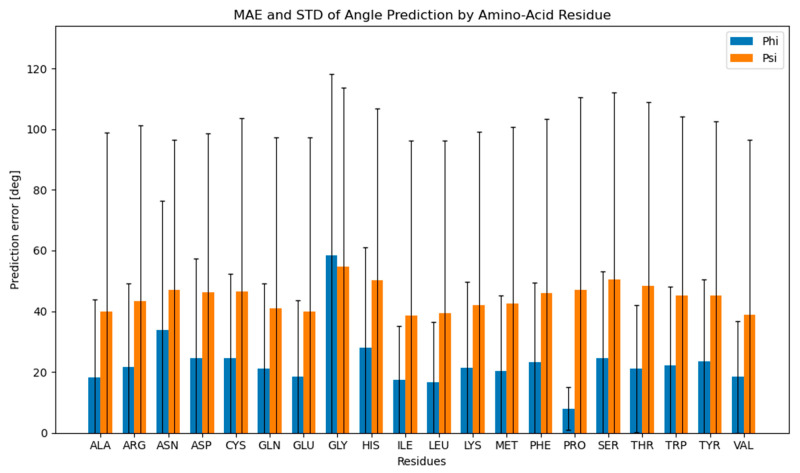
Mean absolute prediction error per amino-acid residue. A mean absolute prediction error and its standard deviation were calculated for each amino-acid residue for ϕ (blue) and ψ (orange) dihedral angles.

For each data row, the mean absolute error (MAE) was determined by utilizing the already-described loss function formula. The mean absolute error and its corresponding standard deviation were then calculated for all 20 amino-acid residues (Figure 3). The resulting mean absolute error was 23.53° for ϕ angles and 44.14° for ψ angles. An important trend can be observed from Figure 3, namely that predictions of phi angles exhibit greater accuracy when compared to psi angles (with the notable exception of glycine).

The poor performance of ψ predictions compared to ϕ predictions originates from the differences in the input data. Namely, as observed beforehand, ϕ and ψ exhibit different distribution patterns, with ϕ presenting a peak at around −90° and an effective distribution of approximately 100°, while ψ possesses two peaks at −110° and 130° with an approximate distribution of 150° (Figure 2). Figure S1 also illustrates that the standard deviation of proline ϕ angles is the smallest (10.93°) in the dataset, as expected, due to the cyclic proline structure, while the standard deviation of glycine ϕ angles is the largest (96.21°), with the average for all remaining amino-acid residues of 39.60° (Table S1). This difference in the measured angle deviations between amino-acid residues corresponds to the pattern observed in the mean absolute prediction error of ϕ angles per amino-acid residue—the proline ϕ angles were the easiest to predict with the mean prediction error of 7.77°, while the small and flexible glycine was the most difficult to predict with the mean prediction error of 58.72°. The differences in the MAE of predictions for ϕ and ψ, therefore, directly correlate with the average standard deviations of real angles in the dataset and follow the structural observations of individual amino-acid residues.

**Figure 4:**
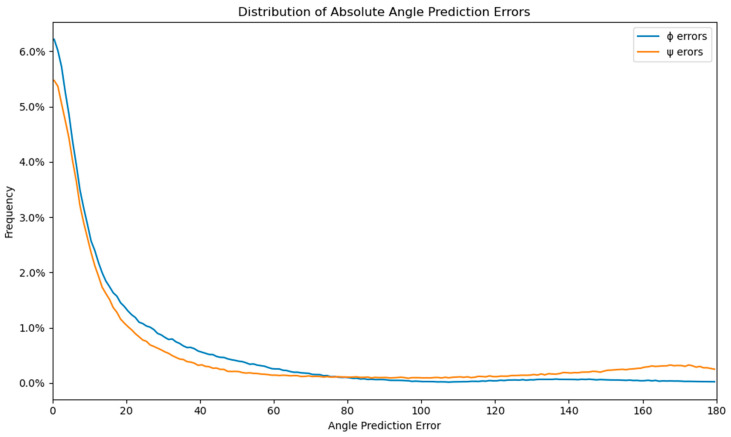
Distribution of the absolute angle prediction errors for ϕ and ψ with a resolution of 1 degree.

Moreover, Figure S2 illustrates that the mean absolute error (MAE) of the current amino-acid residue is not significantly influenced by neighboring amino-acid residues within the sliding window, regardless of its position. However, the presence of the virtual residue “0” (denoting an empty space or the start or end of the protein sequence) significantly impacts the MAE of the current residue. It is known that amino-acid residues near the protein sequence termini often lack a well-defined secondary structure, resulting in a broader distribution of dihedral angles and increased prediction difficulty. The error distributions of ϕ and ψ, shown in Figure 4, exhibit an expected rectangular hyperbola shape (f(x) = n/x), with the majority of errors under 20 degrees (64.85% for ϕ and 57.87% for ψ). The ϕ and ψ error distributions are similar up to 80 degrees, but the number of ψ errors increases from 80 to 180 degrees, representing 23.26% of the error distribution, compared to only 5.78% of the ϕ errors. The errors larger than 179 degrees represent 5.96% of the ψ distribution, about 5 times more than the 1.28% of the same error span for the ϕ distributions. This increase in large errors for the ψ angle is also visible in Figure 2 and Figure 4, which display the comparison of measured and predicted ψ angles. The difference in the large error distribution results in a much larger MAE of ψ angles compared to ϕ angles.

### 2.2. Measured vs. Predicted Values

**Figure 5:**
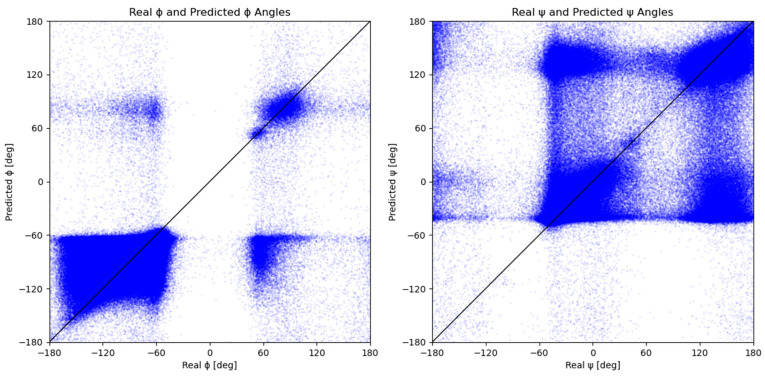
Predicted dihedral angles in relation to the measured dihedral angles. Each blue dot represents one set of measured and predicted ϕ and ψ dihedral angles. The black y = x line represents the ideal distribution, where each predicted angle is equal to its measured counterpart.

The results of Figure 5 show a comparison of measured and predicted dihedral angles, ϕ and ψ. Each blue dot represents a measured-predicted dihedral angle pair. The trend of the distribution of the measured and predicted ϕ and ψ angles from Figure 2 is visible in Figure 5. The graphs in Figure 5 show that the majority of points are divided into two groups that correspond to the major secondary structures and, consequently, to the peaks in Figure 2. Due to the model’s tendency to assign angles that belong to one of the two major secondary structure elements, model predictions result in lower distribution accuracy than individual predictions. This is even more evident from the Figure 5 graph for the ψ dihedral angle since its values are more evenly distributed into two groups.

The discussed groups can be found in Figure 4, located on the graphs’ top left and bottom right edges. A scattered group located around −60° on the x-axis and 70° on the y-axis represents predictions of the ϕ angle that should have been classified as right-handed α-helix, 3_10_-helix, or β-sheet but were instead predicted closer to the left-handed α-helix (Table 1). The other group of mispredicted values is located in the bottom right quadrant, which corresponds to the left-handed α-helix values predicted closer to the right-handed α-helix, 3_10_-helix, or β-sheet in terms of the ϕ angle. An analogous trend can be observed for the graph of measured vs. predicted values of ψ angle, where the resulting scattering is even more prominent due to the existence of two almost equal distributions (Figure 2). Since the peaks of the ψ distribution are almost 180° apart, each mispredicted ψ angle contributes a lot more towards a large MAE than a mispredicted ϕ angle. The Ramachandran plots of the measured and predicted angles (Figure S3) are similar, with well-defined areas for the helices and sheets. The purely predicted Ramachandran plot exhibits higher scattering and a loss of detail when compared to the measured ϕ and ψ angles; however, even a naive one-model prediction can identify general Ramachandran plot trends.

### 2.3. Dihedral Angle Predictability in Amino Acids

Our model permits an examination of the degree to which adjacent amino acid residues influence the structure of a given amino acid residue and the extent of this influence. To observe this effect, we trained and evaluated the same neural network using a varying sliding window input, ranging from 3 to 21 amino acid residues (Table 2). Our preliminary results indicate that the two closest neighboring residues significantly impact the secondary structure, as shown in Table 2. Interestingly, a sliding window of size three already provides a certain level of accuracy in predicting backbone dihedral angles (28.37 for ϕ and 64.09 for ψ in our tests). However, expanding the sliding window to incorporate 21 residues, with 10 amino acid residues on each side, further enhances the model’s accuracy. These findings align with those reported by Chen K. [50], which stated that the formation of a helical structure can be influenced by amino acids situated up to nine positions away in the sequence. Similarly, the formation of coils and strands can be affected by amino acids up to three and six positions away, respectively, suggesting that for optimal secondary structure prediction, a sliding window comprising 19 residues might be most effective.

Furthermore, regions of the Ramachandran plot previously thought to be conformationally uniform, such as the ones corresponding to α-helices or β-structures, can actually be subdivided based on their distinctive conformational propensities, and these propensities are more influenced by the local (ϕ, ψ) angles than by the secondary structure itself [51]. Comparing our test data with the propensities reported in the literature [52], we observed that both our real and predicted amino acid propensities in the Ramachandran space showcase similarities (Table S2). For instance, residues like Ala, Glu, and Gln exhibit a prominent inclination towards specific alpha-helices, while Val, Thr, and Tyr favor beta-sheets, whereas Gly favors coils.

In supplementary information (Figure S2), we illustrate that Pro ϕ dihedral angles are the most straightforward to predict, whereas predicting ϕ or ψ dihedral angles for Gly proves to be the most challenging. This aligns with the observations in Figure S1, which demonstrates the minimal standard deviation among Pro ϕ dihedral angles and the maximum deviation for Gly ϕ dihedral angles. In general, the prediction of dihedral angles is more complex when they are positioned near the beginning or end of a protein sequence. Yet, this does not seem to significantly affect the ψ dihedral angles of Ile and Val. These angles display an MAE better than 30°—an improvement compared to the average ϕ MAE of 44.14°—across a broad range when they occur at the sequence’s start (up to 10 positions). However, this pattern is not observed when these angles are positioned at the sequence’s end.

### 2.4. Three-State Secondary Structure Prediction

Although our model was not trained to predict the discrete elements of the protein secondary structure, it can be repurposed by converting the pairs of dihedral angles ϕ and ψ to the three-state secondary structure elements—helix, sheet, and undesignated. Converting the dihedral angles into secondary structure elements is a challenge in itself because most well-known algorithms utilize atomic coordinates to assign secondary structures; however, our dataset is based on a sliding window of residue labels. For example, the STRIDE [53] algorithm recognizes secondary structure elements using hydrogen bond energy and mainchain dihedral angles, while the DSSP of Kasch and Sander [12] employs hydrogen bonds alone. To assign a three-state secondary structure based solely on dihedral angles ϕ and ψ, three areas were determined based on the Ramachandran plot in VMD software (version 1.9.3.) [54] (Figure S4). The secondary structures were then assigned for both measured and predicted dihedral angles of each amino-acid residue from the test dataset. The accuracy of the predicted secondary structures was subsequently determined by comparing the predicted dihedral angles for each residue in the test dataset to the measured dihedral angles. The accuracy was calculated as the number of correctly predicted residues (347 388) divided by the total number of residues (504 024), resulting in an overall accuracy of 68.9% (Table 1).

This study found that our model most accurately predicted sheet structures at a rate of 73.9%, closely followed by helices at 73.2%. However, the prediction accuracy significantly dropped for undesignated structures, which stood at a mere 46.1%. The high accuracy in sheet structure prediction is attributed to their significant representation in the dataset (44.7%; 225,508), facilitating more effective learning of their angle distributions. Conversely, the wide range of dihedral angles from −180° to 180° and their scarce representation in the dataset (16.9%; 85,391) made undesignated structures particularly challenging to predict. One inherent limitation of our analysis is the conversion of continuous dihedral angles into discrete Q3 labels, which may lead to misclassification of predictions at the boundaries of the secondary structure Ramachandran regions. Additionally, the restriction to Q3 rather than Q8 predictions amalgamated all types of helices, potentially counting incorrect dihedral angle predictions as accurate secondary structure elements. We postulate that for our present input data and given the modest model architecture, a naive classification might yield better results than a regression of individual values. Importantly, the improved accuracy in predicting key secondary protein structure components—helices and sheet structures—offers a simplified, yet effective, approach for secondary structure prediction. This capability paves the way for the creation of a foundation, enabling subsequent refinement for more accurate and comprehensive protein structure modeling.

Upon conducting a per-protein analysis on our test dataset, it was observed that the model exhibited a higher degree of accuracy in predicting helices, as evidenced by the MAE values of 15.13° for phi and 36.58° for psi angle, accordingly, pertaining to all-alpha proteins. Conversely, the predictive accuracy for all-beta proteins was found to be lower, potentially due to their underrepresentation in comparison to all-alpha proteins—they constitute merely 25% of the all-alpha protein count.

Moreover, a correlation study was conducted on eleven further experimental systems. These included a small well-described system (PDB ID: 1CRN [55]; Figure S5), a large system of Salinosporamide A complexed with yeast 20S proteasome (PDB ID: 2FAK [56]; Figure S6), homodimeric hemoglobin (PDB ID: 3QOB [57], Figure S7), a medium-sized system E. Coli DNA gyrase subunit B (PDB ID: 4DUH [58]; Figure S8), IFN alpha8 (PDB ID: 6JHD [59]; Figure S9), crystal structure of HL homo-diabody (PDB ID: 6KR0 [60]; Figure S10), a cryo-EM structure of the human PA200 and PA200-20S complex (PDB ID: 6KWY [61]; Figure S11), S109 in complex with CRM1-Ran-RanBP1 (PDB ID: 6LQ9 [62]; Figure S12), and a structure of NHP D11A.F2 Fab (PDB ID: 6XLZ [63]; Figure S13), as well as both Ala and Val MnSOD models (Figures S14 and S15), based on the X-ray diffraction by Azadmanesh, et al. [64] and studied by Broz et al. [65]. All model predictions exhibit MAE values similar to the test dataset predictions, ranging from 12.24°to 28.26° for ϕ errors and from 16.70° to 77.66° for ψ errors (Table S3), and the I model could generally classify the correct secondary structure.

## 3. Materials and Methods

### 3.1. Dataset Preparation

The dataset was created by extracting proteins from the PISCES [66] database as of February 2023 (Figure S16), which represents a public server for culling sets of protein sequences from the protein data bank (PDB) [67] by sequence identity and structural quality criteria. This database was selected for its ability to provide high-quality lists compared to servers that use BLAST, which often overestimate sequence identity by aligning only well-conserved fragments (alternatives are CoDNas datasets). The criteria used to extract the proteins from PISCES were R-free < 0.25 and protein length ranging from 40 to 10,000 amino acid residues, resulting in a total of 64,220 protein chains. Additional filters were then applied to select models with a resolution of <2.5 Å, one chain per PDB entry, and no missing amino-acid residues, resulting in a final dataset of 20,605 protein chains. To prepare the dataset for analysis, the coordinates of each residue were converted into backbone dihedral angles ϕ and ψ using the Biopython 1.75 Bio.PDB package [68] and Python programming package version 3.10 [69]. The analysis excluded side chains and only considered the first listed amino-acid residue in cases where multiple residues were present due to mutation studies.

Our method employed a sliding window of size 21 for each row of data, which comprises the current amino-acid residue and the 10 residues preceding and following it. This choice was motivated by the trade-off between model accuracy and computational efficiency. As demonstrated in Table 2, while larger window sizes slightly increased accuracy, they also substantially extended the training duration due to the increase in data complexity. At a window size of 21, the accuracy gains practically diminished while computational time escalated.

**Figure 6:**
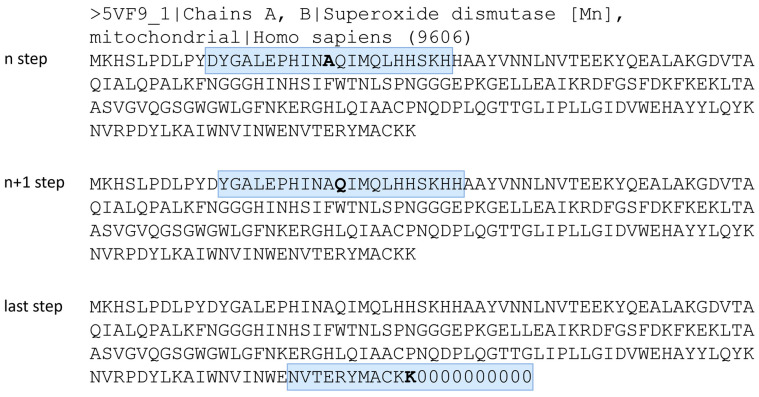
A schematic representation of the amino-acid residue sliding window, using the FASTA sequence of the 5VF9 [64] model. The sliding window consists of 10 amino-acid residues before and 10 amino-acid residues after the current amino-acid residue, shifting the window by one amino-acid residue to the right at each step. Virtual residues denoted by “0” were added to the start and end of the sequence to define the sliding window in these regions. The current amino-acid residue is indicated with a bold letter, while the sliding window is highlighted in light blue.

At the start and end of the protein sequences, where a sliding window of size 21 could not be defined, a virtual amino-acid residue denoted as “0” was introduced. For example, a sliding window of size 21 for a mitochondrial human manganese superoxide dismutase is presented in Figure 6.

In this work, we applied one-hot encoding to the one-letter amino-acid codes using the get_dummies function of the Pandas 1.3.5 library. Each amino-acid residue in the sliding window was encoded into a 20 × 1 vector, yielding 420 independent variables (21 × 20 = 420). Notably, the 21st virtual amino-acid residue we introduced to denote empty spaces at sequence beginnings or ends was represented with a null vector rather than with an additional 20 × 1 vector to optimize computational efficiency. This strategy reduced the potential number of independent variables from 438 to 420 without information loss. The resulting dataset comprised 5,040,244 rows and 422 columns, with the 420 columns representing the one-hot encoded amino-acid residues (the independent variables) and the 2 columns indicating the real ϕ and ψ angles (the dependent variables). This dataset was split into training (80%), validation (10%), and testing (10%) datasets using the train_test_split function in the Tensorflow 2.11.0 package [70].

### 3.2. Neural Network

**Figure 7:**
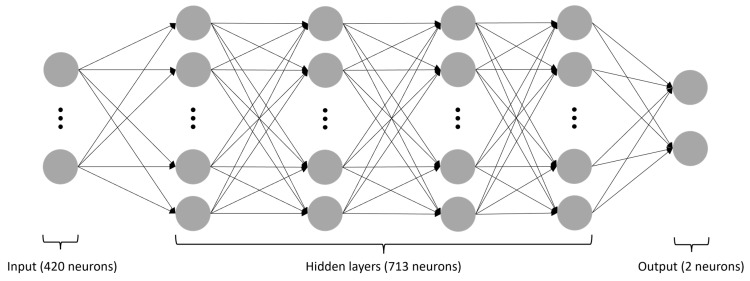
The structure of the applied neural network. The gray circles symbolize individual neurons, while the arrows indicate connections from the output of one artificial neuron to the input of another. This network consists of four hidden layers, with the input layer containing 420 neurons, all four hidden layers 713 neurons each, and the output layer 2 neurons. All layers, except the last one, utilize the ReLU activation function.

In this study, a sequential neural network was utilized to solve a regression problem. The network consisted of an input layer with 420 neurons, four hidden layers with 713 neurons, and an output layer with two neurons (Figure 7). All hidden layers employed the rectified linear unit (ReLU) activation function, while the input and output layers utilized a linear activation function. The network was implemented using the Keras library [71] and Adadelta optimizer [72] with default settings. Network topology was defined and optimized using the Optuna library (https://optuna.org/; accessed on 28 August 2023). In this study, the Adadelta optimizer was selected for training the sequential neural network due to its adaptability, efficiency, and proven success in deep learning, as it forms an adaptive learning rate optimization algorithm that does not require the specification of a fixed learning rate or momentum parameter. The learning rate was set to 0.5, and the training was stopped after four iterations without the validation loss function improvement. The model was trained on four AMD EPYC 7402 24-Core processors.

### 3.3. Loss Function

**Figure 8:**
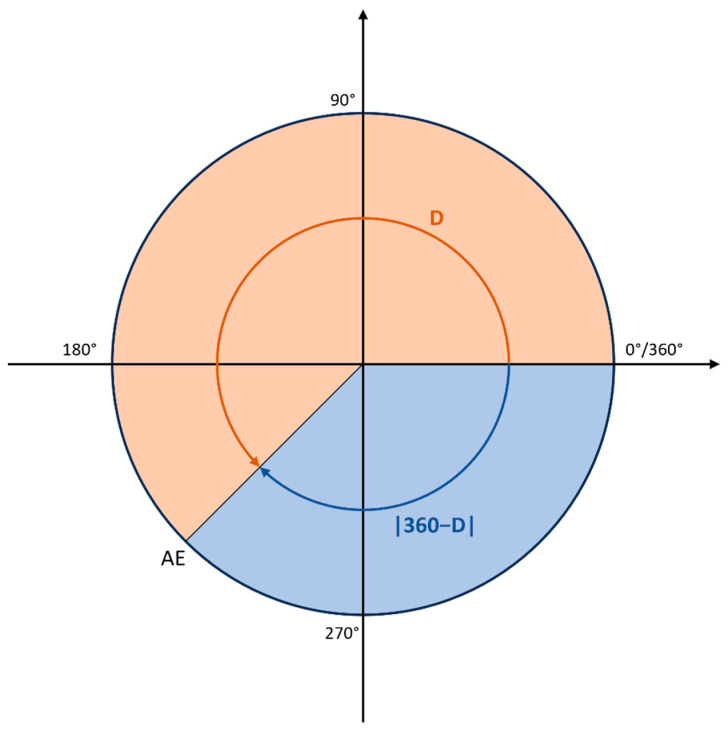
The schematic representation of the custom loss function. For illustrative purposes, the real angle is set at 0 degrees, while the predicted angle is 245 degrees. In this case, D is 245°, while |360° − D| is 115°. The custom loss function adopts the minimum value between D and |360° − D|, which in this instance is 115°. This methodology ensures that all calculated errors are confined within the appropriate range of [0, 180°].

The custom loss function was used instead of root mean squared error (RMSE) or MAE because it addresses the periodicity of angles. The function calculates the absolute error for each residue using Equation (1):

AE = min(D, |360 − D|),(1)

where D = |P − A|, P is the predicted angle, and A is the measured angle for an amino-acid residue (Figure 8). This equation ensures that errors near the wrap-around point of 360 degrees are handled appropriately. Namely, due to the periodic nature of angles, the minimum error is 0 degrees and the maximum error is 180 degrees. The mean of these absolute errors is then used as the loss function to train, evaluate, and test the neural network. This approach is consistent with previous studies (SAP [46] and SAP4SS [47]) and is used during all stages of the analysis.

### 3.4. Optimization

The Bayesian optimization method [73] was constructed using the create_study function in the Optuna hyperparameter optimization framework [74]. While it offers a wealth of sampling and pruning background algorithms, the default settings were used—this encompassed the tree-structured Parzen estimator (TPE) sampler [75] and MedianPruner for pruning. The minimization method was employed to optimize the number of layers and neurons per layer. Bayesian optimization facilitated a more effective exploration of the hyperparameter space through modeling the objective function and employing a probabilistic strategy to maintain a balance between exploration and exploitation during the parameter tuning phase.

**Figure 9:**
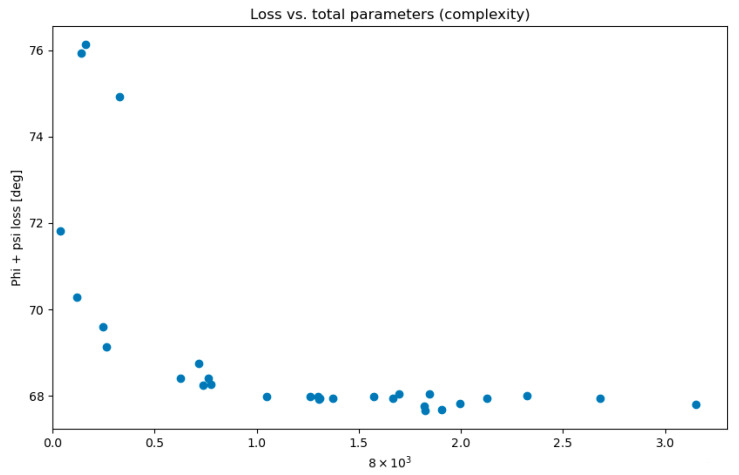
Total loss function (ϕ + ψ) in relation to the number of parameters of the neural network. The total number of parameters in a sequential neural network was calculated by adding the product of the number of neurons in each layer with the number of neurons in the following layer for all layers. This represents the total number of connection weights between neurons in adjacent layers, which yields a single number indicative of the network’s size and complexity.

The possible hidden layer count was established within a range of one to five, while the neuron count was determined to be between 26 and 840, representing 1/16th and twice the number of the input features, respectively. The optimization procedure, aimed at minimizing the sum of the ϕ and ψ loss functions, was carried out over 30 trials. Figure 9 illustrates that the loss function stopped improving beyond a certain complexity of our neural network. The complexity of a neural network is also directly correlated with the training duration.

In addition to our primary dual-output neural network, we explored potential improvements by optimizing two separate neural networks, each featuring a single output neuron dedicated to predicting either the ϕ or ψ angle. However, as shown in Tables S1–S3, neither approach provided superior accuracy (Figure S17). The two-neuron approach traded off accuracy between the angles, reducing one to minimize the sum of both. Therefore, while either method could be utilized when accuracy is the primary concern, we propose that the dual-output neural network, predicting both angles simultaneously, presents a time-efficient and robust solution. The results can also easily be coupled to further processing steps if needed.

### 3.5. Predicted Outputs

The neural network has two outputs, representing both ϕ and ψ angles. Hence, only one model was trained for the simultaneous prediction of ϕ and ψ angles. Each pair of ϕ and ψ was associated with one vector with dimensions of 420 × 1, accounting for both angles within one sliding window combination. The angles were managed directly, accommodating their periodicity (−180° to 180°) within the custom loss function of the DNN applied, eliminating the need for using sine and cosine ratios.

## 4. Conclusions

Our research provides an in-depth analysis of a neural network model that employs a relatively small sliding window in relation to the entire protein length. This approach facilitates our model’s ability to predict dihedral angles without the need for detailed descriptors or the involvement of molecular dynamics simulations. While this streamlined methodology offers clarity in terms of computational understanding, it introduces limitations. Particularly, our model does not effectively capture intra-protein interactions between amino-acid residues located more than ten residues apart, and consequently, it might miss essential molecular interactions such as disulfide bonds, hydrophobic and hydrophilic interactions, hydrogen bonds, and salt bridges.

Despite these challenges, our model represents a perspective application for ab initio structure prediction and the preliminary stages of refinement by providing a basic yet informative representation of the protein backbone structure. Through our evaluations, the model has shown the ability to identify Ramachandran trends and categorize dihedrals into secondary structure elements effectively. While it is not designed to compete directly with advanced, complete folding workflows, its potential within a broader protein folding framework is evident. It is worth noting that many leading protein prediction models integrate multiple simpler models to produce initial structures, which are then refined. Our FCNN model can be envisioned as capable of integrating within such a layered prediction system or being supplemented with additional data to enhance its predictive capabilities.

The central theme of our research has been the exploration of the relationship between model complexity and predictive accuracy. The results from our FCNN model suggest that a model built on a 21-residue primary sequence can achieve notable accuracy in predicting ϕ and ψ dihedral angles. This emphasizes the importance of simplicity in model selection and development—an aspect that sometimes gets overlooked in the current landscape of multi-layered prediction systems. Through our work, we aim to highlight the benefits of balancing performance with computational simplicity. In conclusion, our study encourages the scientific community to reflect on the role of model complexity in determining predictive outcomes. It offers researchers insights into selecting the right model structure and computational approach for their protein folding investigations. In the spirit of open science, our complete work, including the datasets used, is freely available at: https://github.com/maticbroz/phi_psi_prediction_FCNN (accessed on).

## Figures and Tables

**Table 1:** The portion of the real helix, sheet, and undesignated secondary structures predicted as either helix, sheet, or undesignated secondary structures.

SS Label	Helix (Predicted)	Sheet (Predicted)	Undesignated (Predicted)
Helix (real)	73.2% (141,380)	16.0% (30,837)	10.8% (20,908)
Sheet (real)	14.9% (33,535)	73.9% (166,673)	11.2% (25,300)
Undesignated (real)	27.1% (23,151)	26.8% (22,905)	46.1% (39,335)

**Table 2:** The correlation between the sliding window size, the accuracy of the model, and the training duration.

Sliding Window	Phi MAE [deg]	Psi MAE [deg]	Epoch Duration [s]
3	28.37	64.09	57
7	25.67	53.36	102
11	24.51	48.98	130
15	23.96	46.74	155
21	23.53	44.14	210

## Data Availability

All data is contained within the article, supplementary material, and the provided GitHub repository.

## Supplementary Materials

The following supporting information can be downloaded at: https://www.mdpi.com/article/10.3390/molecules28207046/s1, Table S1: Exact values of standard deviations of real ϕ and ψ angles in the train and test datasets; Table S2: Real and predicted amino acid conformational propensites of the test dataset; Table S3: Per-model mean absolute errors of the predicted phi and psi dihedral angle of our model com-pared to an online tool DISSpred; Figure S1: A bar graph of standard deviations of real ϕ and ψ angles in the entire dataset by amino-acid residue; Figure S2: Mean absolute error of the current amino-acid residue in relation to the amino-acid residues at different positions of the sliding window for the ϕ (left) and ψ (right) dihedral angles; Figure S3: Ramachandran plots of actual and predicted dihedral angles; Figure S4: Secondary structure areas in the Ramachandran plot. The areas represent secondary structure elements: helices (red), sheets (purple), and undesignated (white). The areas are enclosed by the following points: helix = [(−180.0, −34.9), (−164.3, −42.9), (−133.0, −42.9), (−109.5, −32.2), (−106.9, −21.4), (−44.3, −21.4), (−44.3, −71.1), (−180.0, −71.1)] AND [(62.6, 14.7), (62.6, 96.7), (45.6, 79.2), (45.6, 26.8), (62.6, 14.7)]; sheet = [(−180.2, 42.9), (−140.8, 16.1), (−86.0, 16.1), (−74.3, 45.6), (−74.3, 72.5), (−44.3, 102.0), (−44.3, 161.1), (−46.9, 179.9), (−180, 180)] AND [(−180.0, −163.8), (−75.6, −163.8), (−46.9, −180.0), (−180.0, −180.0)]; Figure S5: Real and predicted ϕ and ψ angles for the protein structure PDB ID: 1CRN (ϕ error = 24.91°; ψ error = 80.86°); Figure S6 Real and predicted ϕ and ψ angles for the protein structure PDB ID: 2FAK (ϕ error = 25.71°; ψ error = 41.96°); Figure S7 Real and predicted ϕ and ψ angles for the protein structure PDB ID: 3QOB (ϕ error = 17.76°; ψ error = 36.43°); Figure S8 Real and predicted ϕ and ψ angles for the protein structure PDB ID: 4DUH (ϕ error = 29.86°; ψ error = 48.16°); Figure S9 Real and predicted ϕ and ψ angles for the protein structure PDB ID: 6JHD (ϕ error = 24.57°; ψ error = 39.91°); Figure S10 Real and predicted ϕ and ψ angles for the protein structure PDB ID: 6KR0 (ϕ error = 12.27°; ψ error = 16.10°); Figure S11 Real and predicted ϕ and ψ angles for the protein structure PDB ID: 6KWY (ϕ error = 22.78°; ψ error = 42.43°); Figure S12 Real and predicted ϕ and ψ angles for the protein structure PDB ID: 6LQ9 (ϕ error = 18.83°; ψ error = 40.67°); Figure S13 Real and predicted ϕ and ψ angles for the protein structure PDB ID: 6XLZ (ϕ error = 17.45°; ψ error = 23.29°); Figure S14 Real and predicted ϕ and ψ angles for the protein structure of MnSOD_ALA (ϕ error = 26.82°; ψ error = 51.12°); Figure S15 Real and predicted ϕ and ψ angles for the protein structure of MnSOD_VAL (ϕ error = 26.41°; ψ error = 50.44°); Figure S16: The process of generating output, which involves accessing the PISCES and RCSB datasets, converting the coordinates to dihedral angles, and transforming the sequence into a sliding window format; Figure S17: Training and validation loss function values during the ϕ and ψ model training.

## Abbreviations

7PCP	7 physicochemical properties
AMD	Advanced Micro Devices
ASA	accessible surface area
B	isolated β-bridge
BAP	protein backbone angle predictions
BLAST	Basic Local Alignment Search Tool
BRNN	bidirectional recurrent neural network
C	carbon atom ooil
CASP	Critical Assessment of protein Structure Prediction
CNN	convolutional neural network
CoDNas	Conformational Diversity of Native State
Cα	alpha carbon atom
DNN	deep neural network
E	parallel/anti-parallel β sheet conformation
E	sheet
FCNN	fully connected neural network
FM	free modeling
G	3_10_ helix
H	α-helix
HHBlits	HMM-HMM-based lightning-fast iterative sequence search
HMM	hidden Markov model
I	π-helix
LSTM	long short-term memory
LSTM-BRNNs	long short-term memory and bidirectional recurrent neural networks
MAE	mean absolute error
MD	molecular dynamics
MTS	mitoargetingtargetting sequence
MnSOD	manganese superoxide dismutase
N	nitrogen atom
PDB	Protein Data Bank
PISCES	Protein sequence culling server
PSP	protein structure prediction
PSSM	position-specific scoring matrix
PSSP	protein secondary structure prediction
Q3	three-state model
Q8	eight-state model
R-free	Free R-value
RMSE	root mean squared error
ReLU	Rectified Linear Unit
ResNets	Residual Networks
ResNets	residual networks
S	bend
SAP	structure analysis and prediction
SNP	single nucleotide polymorphism
SS	secondary structure
SSPro	Secondary Structure Prediction
SVM	support vector machine
T	turn
Å	Angstrom
τ	tao
ψ	psi dihedral angle
ω	omega dihedral angle
ϕ	phi dihedral angle
