# Supplementary material for: Naive Prediction of Protein Backbone Phi and Psi Dihedral Angles Using Deep Learning
Source: Molecules. 2023 Oct 12;28(20):7046. doi: 10.3390/molecules28207046 (PMC10609058; doi:10.3390/molecules28207046)
Supplement: Supplementary file 1 [file molecules-28-07046-s001.zip › molecules-2615424-supplementary-done.pdf]

molecules

MDPI

Supporting Information

Naive Prediction of Protein Backbone Phi and Psi Dihedral Angles Using Deep Learning

Matic Broz [1], Marko Jukić [1,2,,3*] and Urban Bren [1,2,3,*]

1 Faculty of Chemistry and Chemical Engineering, University of Maribor, Smetanova ulica 17, SI-2000 Maribor, Slovenia; matic.broz@um.si; marko.jukic@um.si; urban.bren@um.si;
2 Faculty of Mathematics, Natural Sciences and Information Technologies, University of Primorska, Glagoljaška ulica 8, SI-6000 Koper, Slovenia;
3 Institute of Environmental Protection and Sensors, Beloruska ulica 7, SI-2000 Maribor, Slovenia;
* To whom the correspondence should be addressed; marko.jukic@um.si and urban.bren@um.si

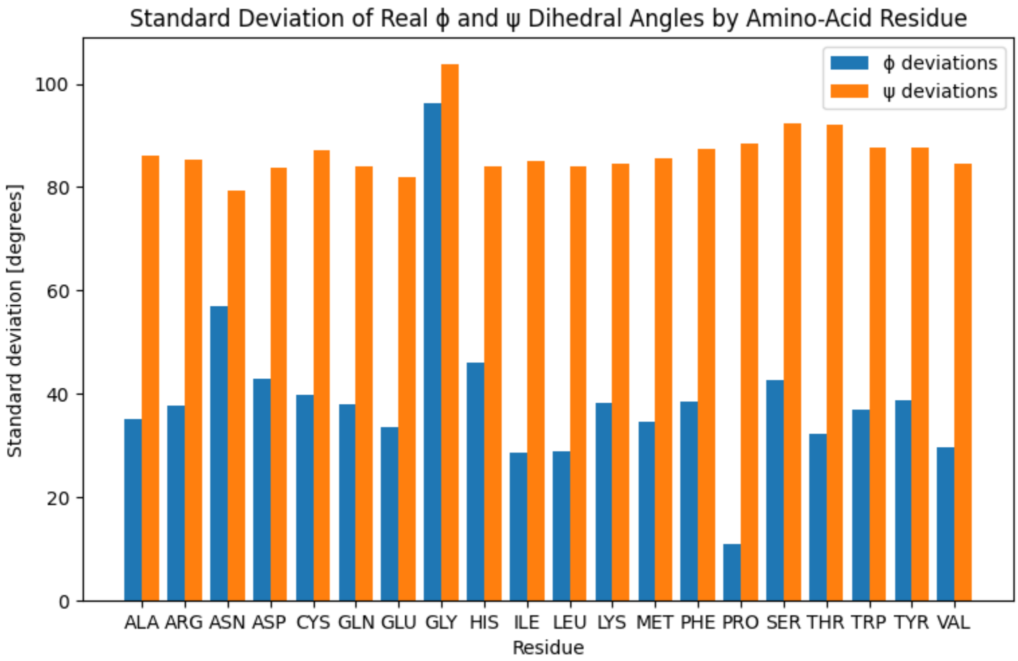

Figure S1. A bar graph of standard deviations of real ϕ and ψ angles in the entire dataset by amino-acid residue.

Table S1. Exact values of standard deviations of real ϕ and ψ angles in the train and test datasets.

Amino-acid residue	φ	Ψ
**ALA**	35.60	86.03
**ARG**	38.43	85.36
**ASN**	57.24	79.45
**ASP**	43.04	82.89
**CYS**	40.57	87.62
**GLN**	37.42	84.01
**GLU**	33.40	81.83
**GLY**	**96.21**	**103.41**
**HIS**	45.06	84.18
**ILE**	28.64	85.19
**LEU**	28.90	84.04
**LYS**	38.09	84.24

**MET**	34.79	85.69
**PHE**	38.23	87.17
**PRO**	**10.93**	88.23
**SER**	42.12	92.21
**THR**	31.99	92.51
**TRP**	36.87	87.83
**TYR**	38.62	87.47
**VAL**	29.85	84.83

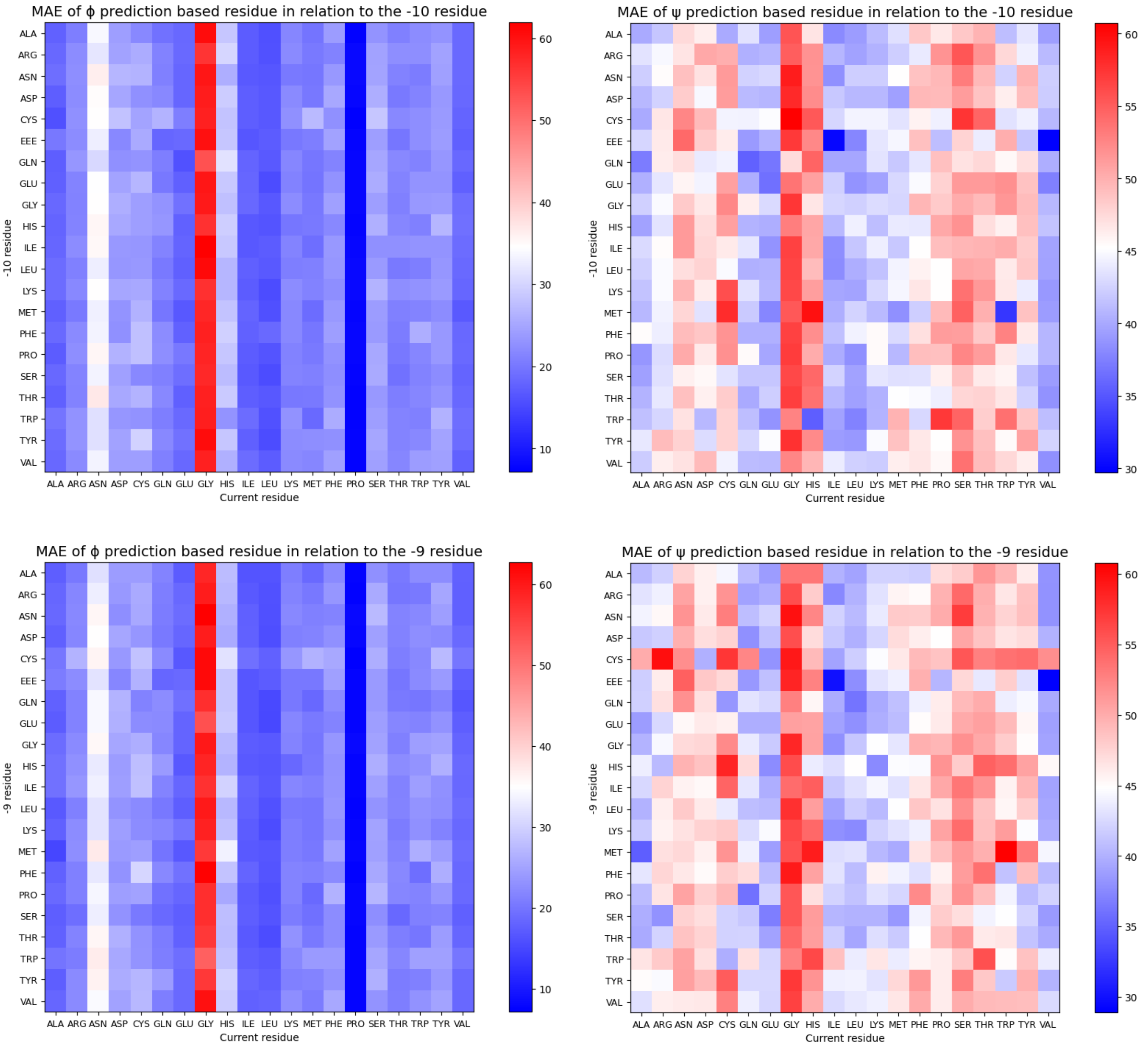

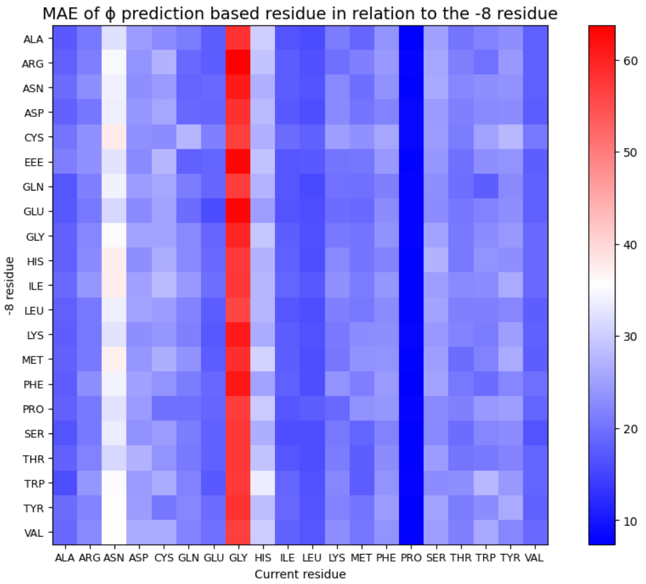
MAE of ϕ prediction based residue in relation to the -8 residue
-8 residue
Current residue
ALA ARG ASN ASP CYS GLN GLU GLY HIS ILE LEU LYS MET PHE PRO SER THR TRP TYR VAL
ALA ARG ASN ASP CYS EEE GLN GLU GLY HIS ILE LEU LYS MET PHE PRO SER THR TRP TYR VAL
10 20 30 40 50 60

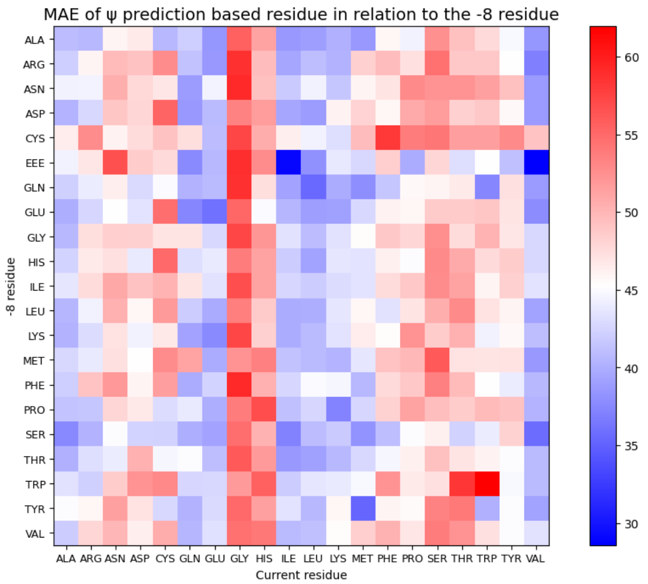
MAE of ψ prediction based residue in relation to the -8 residue
-8 residue
Current residue
ALA ARG ASN ASP CYS GLN GLU GLY HIS ILE LEU LYS MET PHE PRO SER THR TRP TYR VAL
ALA ARG ASN ASP CYS EEE GLN GLU GLY HIS ILE LEU LYS MET PHE PRO SER THR TRP TYR VAL
30 35 40 45 50 55 60

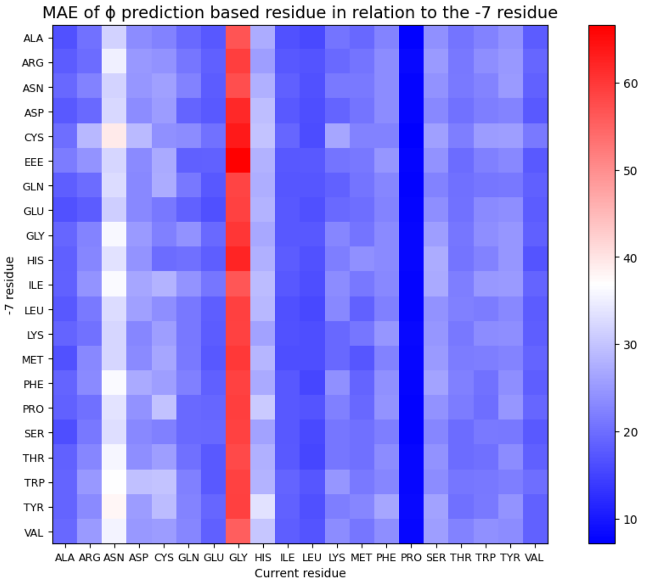
MAE of ϕ prediction based residue in relation to the -7 residue
-7 residue
Current residue
ALA ARG ASN ASP CYS GLN GLU GLY HIS ILE LEU LYS MET PHE PRO SER THR TRP TYR VAL
ALA ARG ASN ASP CYS EEE GLN GLU GLY HIS ILE LEU LYS MET PHE PRO SER THR TRP TYR VAL
10 20 30 40 50 60

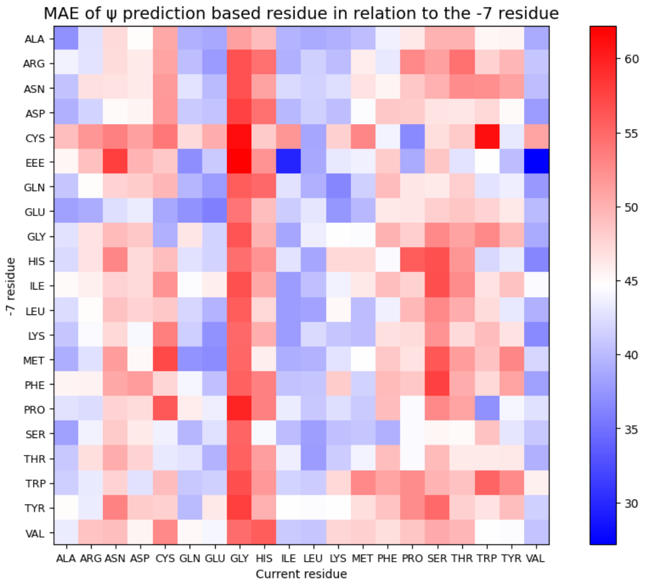
MAE of ψ prediction based residue in relation to the -7 residue
-7 residue
Current residue
ALA ARG ASN ASP CYS GLN GLU GLY HIS ILE LEU LYS MET PHE PRO SER THR TRP TYR VAL
ALA ARG ASN ASP CYS EEE GLN GLU GLY HIS ILE LEU LYS MET PHE PRO SER THR TRP TYR VAL
30 35 40 45 50 55 60

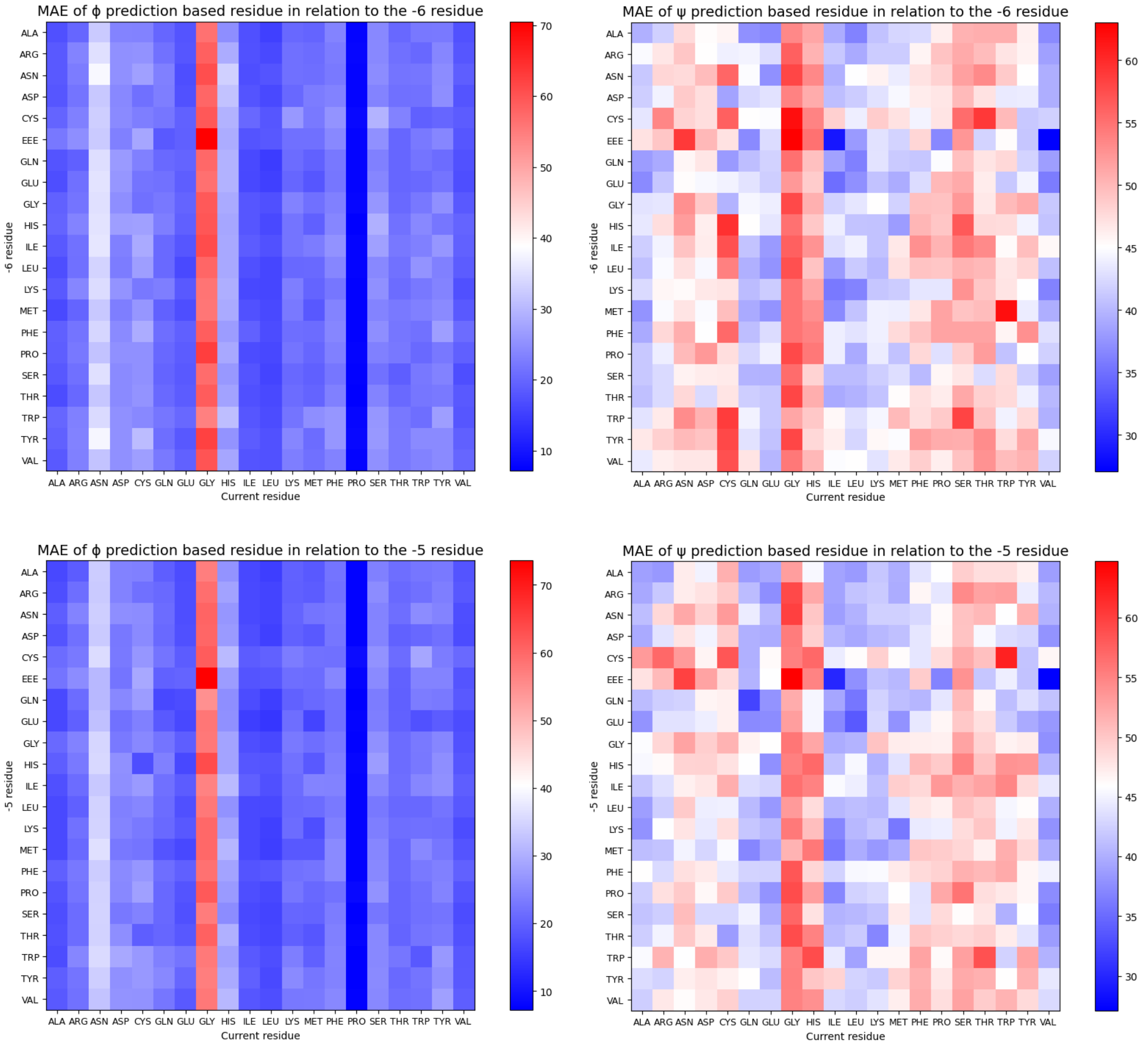
MAE of ϕ prediction based residue in relation to the -6 residue
MAE of ψ prediction based residue in relation to the -6 residue
MAE of ϕ prediction based residue in relation to the -5 residue
MAE of ψ prediction based residue in relation to the -5 residue
-6 residue
-5 residue
Current residue
ALA ARG ASN ASP CYS GLN GLU GLY HIS ILE LEU LYS MET PHE PRO SER THR TRP TYR VAL
ALA ARG ASN ASP CYS EEE GLN GLU GLY HIS ILE LEU LYS MET PHE PRO SER THR TRP TYR VAL

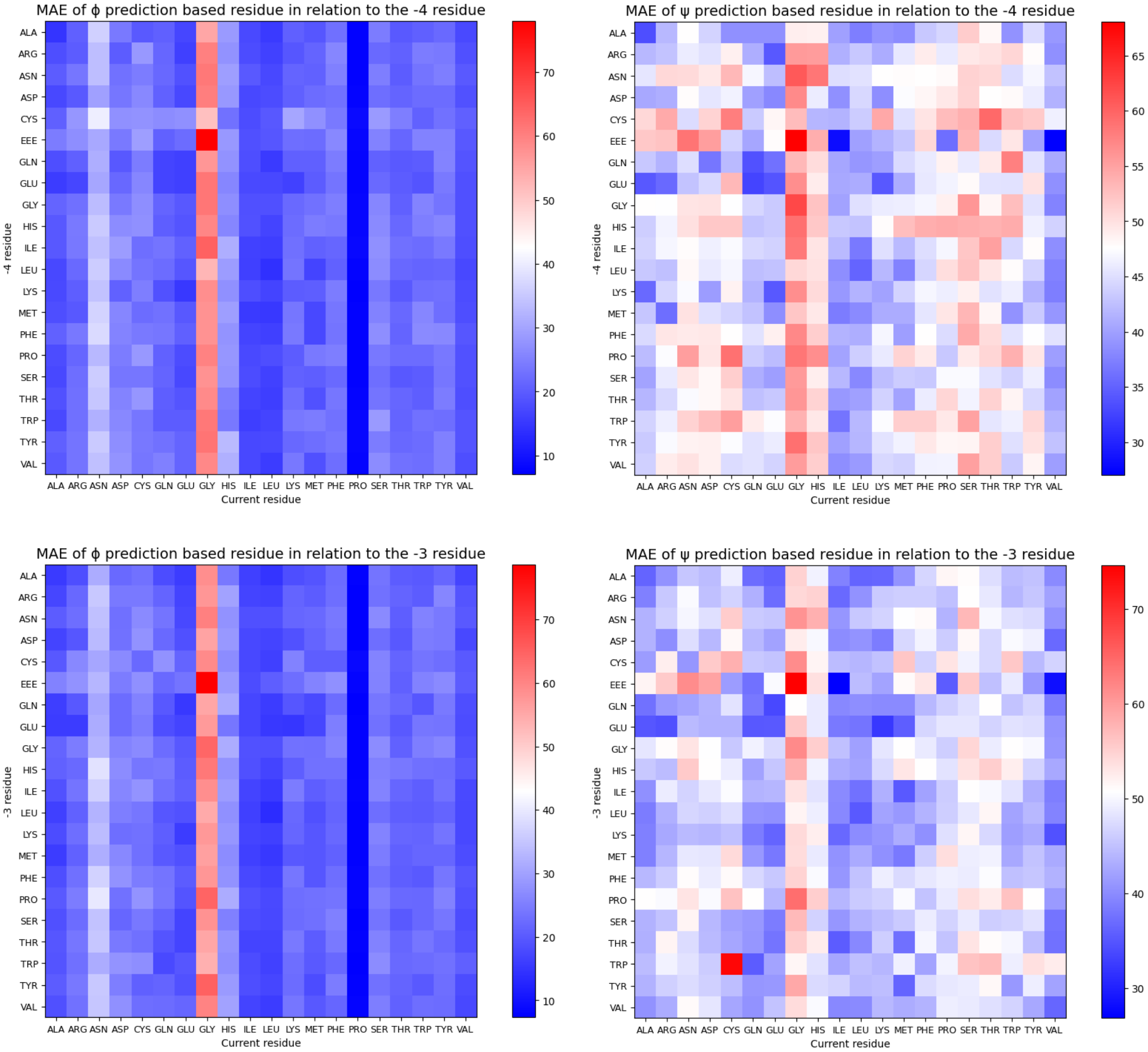
MAE of ϕ prediction based residue in relation to the -4 residue
MAE of ψ prediction based residue in relation to the -4 residue
MAE of ϕ prediction based residue in relation to the -3 residue
MAE of ψ prediction based residue in relation to the -3 residue
-4 residue
-3 residue
Current residue
ALA ARG ASN ASP CYS EEE GLN GLU GLY HIS ILE LEU LYS MET PHE PRO SER THR TRP TYR VAL
ALA ARG ASN ASP CYS GLN GLU GLY HIS ILE LEU LYS MET PHE PRO SER THR TRP TYR VAL
10 20 30 40 50 60 70
30 35 40 45 50 55 60 65
30 40 50 60 70

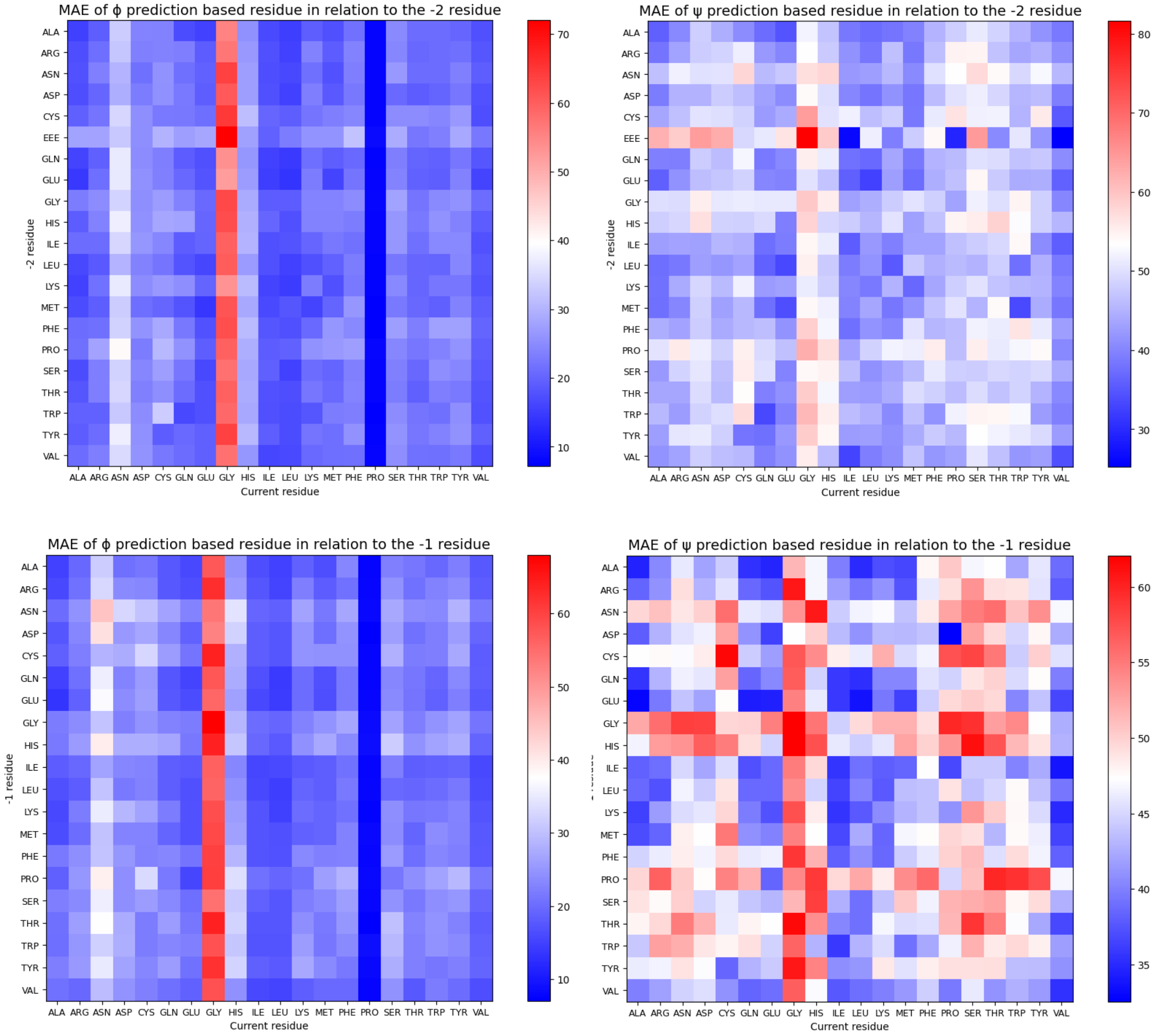
MAE of ϕ prediction based residue in relation to the -2 residue
MAE of ψ prediction based residue in relation to the -2 residue
MAE of ϕ prediction based residue in relation to the -1 residue
MAE of ψ prediction based residue in relation to the -1 residue
-2 residue
-1 residue
Current residue
ALA ARG ASN ASP CYS EEE GLN GLU GLY HIS ILE LEU LYS MET PHE PRO SER THR TRP TYR VAL
ALA ARG ASN ASP CYS GLN GLU GLY HIS ILE LEU LYS MET PHE PRO SER THR TRP TYR VAL
10 20 30 40 50 60 70
30 40 50 60 70 80
35 40 45 50 55 60

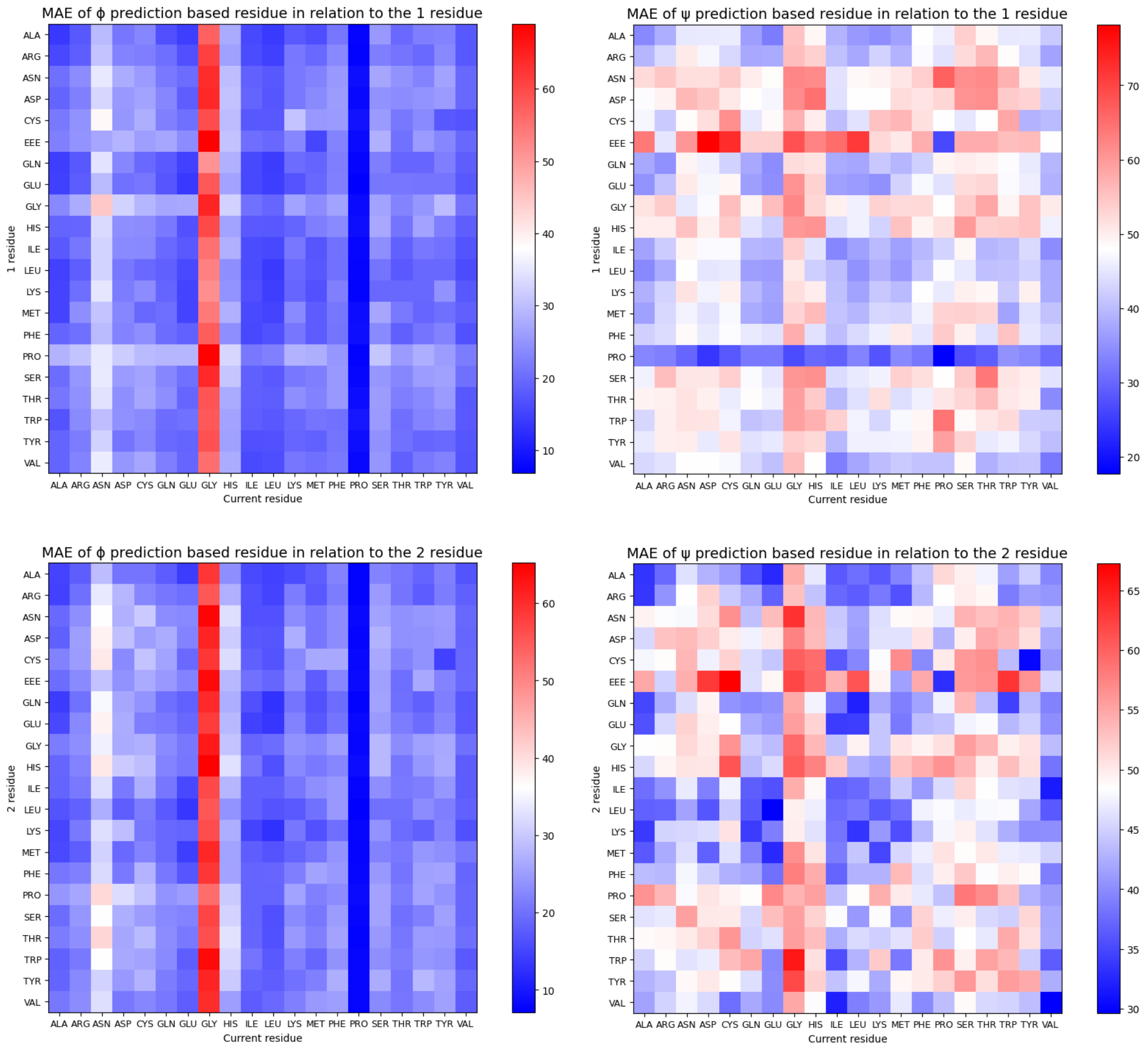
MAE of ϕ prediction based residue in relation to the 1 residue
MAE of ψ prediction based residue in relation to the 1 residue
MAE of ϕ prediction based residue in relation to the 2 residue
MAE of ψ prediction based residue in relation to the 2 residue
1 residue
2 residue
Current residue
ALA ARG ASN ASP CYS EEE GLN GLU GLY HIS ILE LEU LYS MET PHE PRO SER THR TRP TYR VAL
ALA ARG ASN ASP CYS GLN GLU GLY HIS ILE LEU LYS MET PHE PRO SER THR TRP TYR VAL

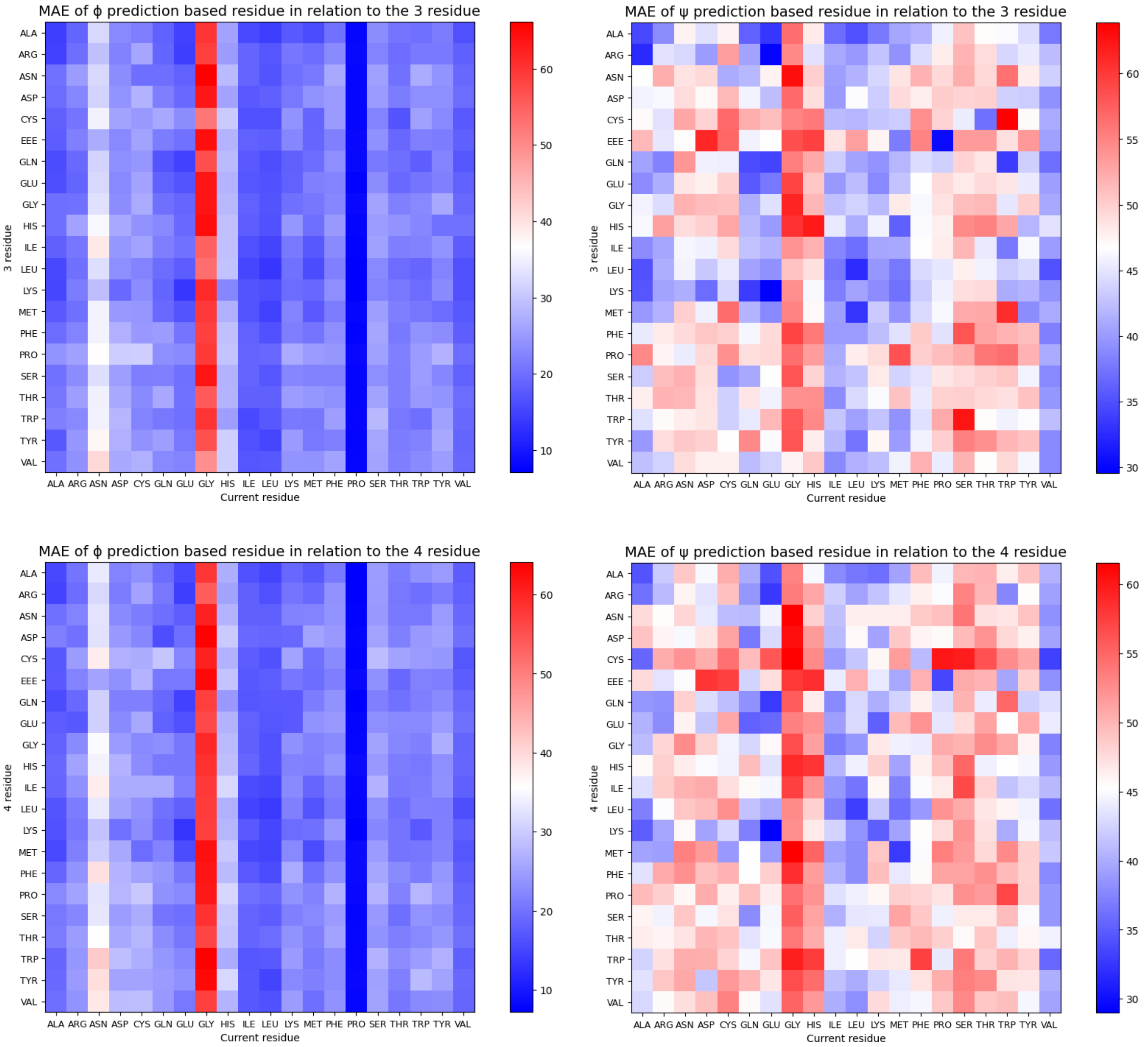
MAE of ϕ prediction based residue in relation to the 3 residue
MAE of ψ prediction based residue in relation to the 3 residue
MAE of ϕ prediction based residue in relation to the 4 residue
MAE of ψ prediction based residue in relation to the 4 residue
3 residue
4 residue
Current residue
ALA ARG ASN ASP CYS EEE GLN GLU GLY HIS ILE LEU LYS MET PHE PRO SER THR TRP TYR VAL
ALA ARG ASN ASP CYS GLN GLU GLY HIS ILE LEU LYS MET PHE PRO SER THR TRP TYR VAL
10 20 30 40 50 60
30 35 40 45 50 55 60

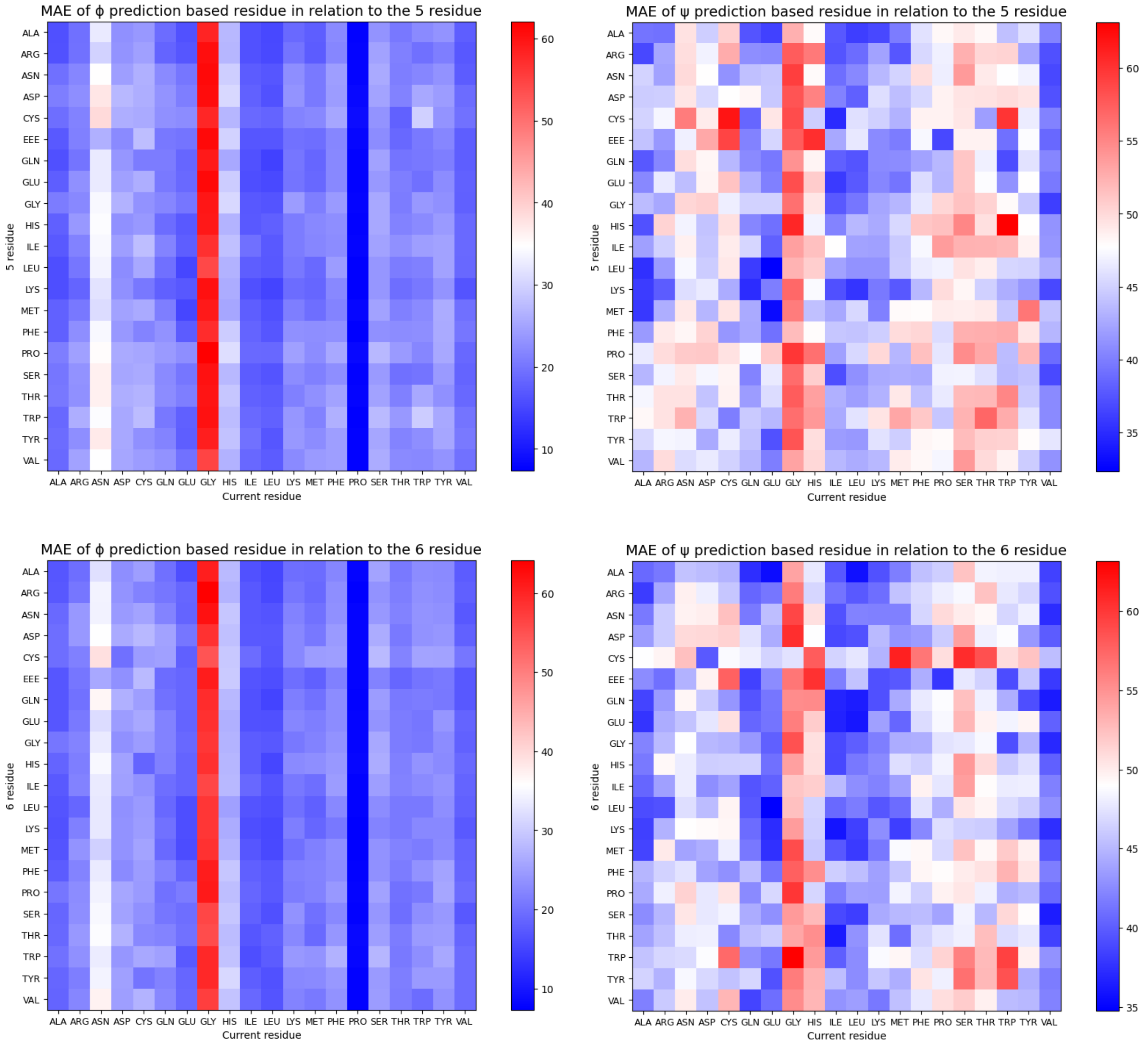
MAE of ϕ prediction based residue in relation to the 5 residue
MAE of ψ prediction based residue in relation to the 5 residue
MAE of ϕ prediction based residue in relation to the 6 residue
MAE of ψ prediction based residue in relation to the 6 residue
5 residue
6 residue
Current residue
ALA ARG ASN ASP CYS EEE GLN GLU GLY HIS ILE LEU LYS MET PHE PRO SER THR TRP TYR VAL
ALA ARG ASN ASP CYS GLN GLU GLY HIS ILE LEU LYS MET PHE PRO SER THR TRP TYR VAL
10 20 30 40 50 60
35 40 45 50 55 60

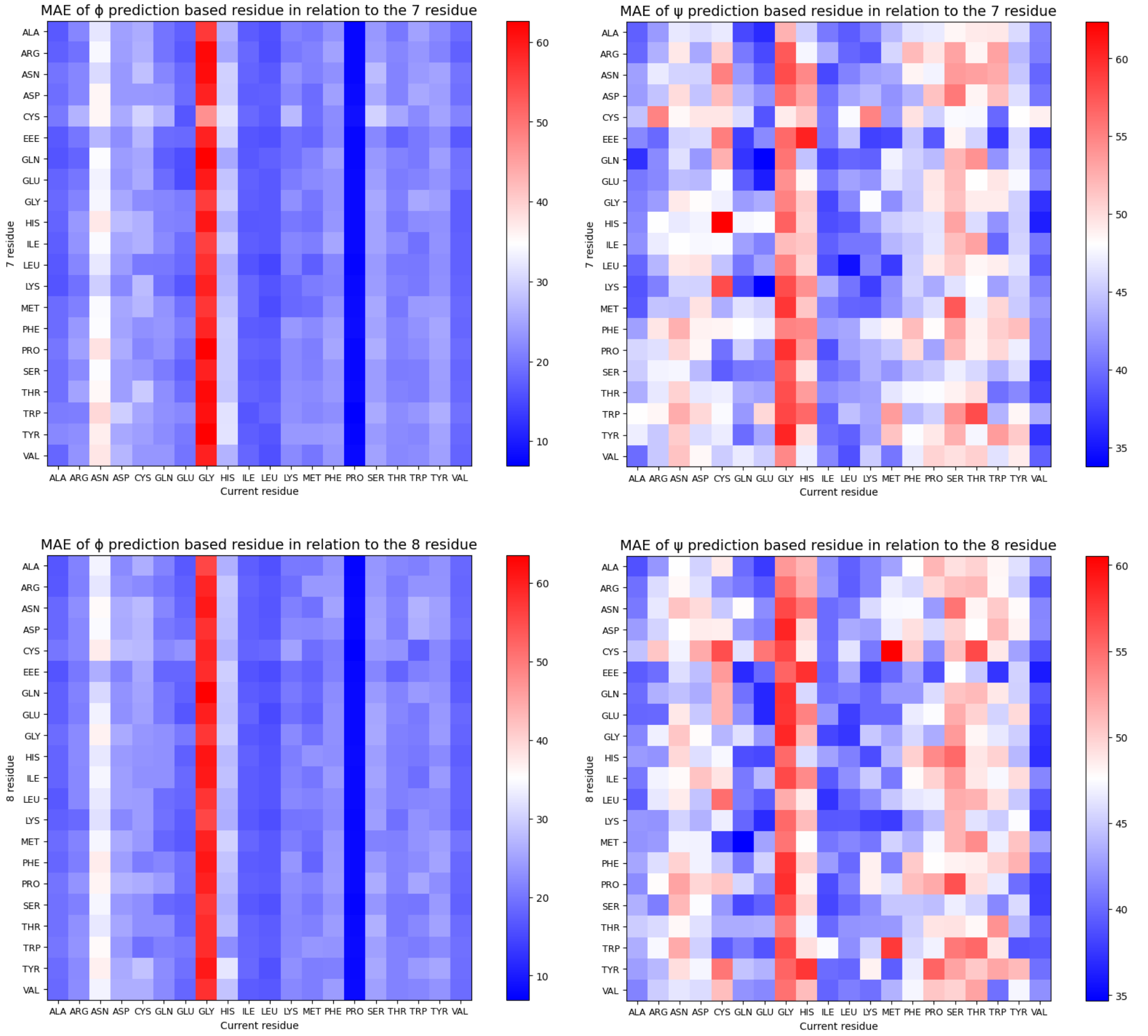
MAE of ϕ prediction based residue in relation to the 7 residue
MAE of ψ prediction based residue in relation to the 7 residue
MAE of ϕ prediction based residue in relation to the 8 residue
MAE of ψ prediction based residue in relation to the 8 residue
7 residue
8 residue
Current residue
ALA ARG ASN ASP CYS GLN GLU GLY HIS ILE LEU LYS MET PHE PRO SER THR TRP TYR VAL
ALA ARG ASN ASP CYS EEE GLN GLU GLY HIS ILE LEU LYS MET PHE PRO SER THR TRP TYR VAL
10 20 30 40 50 60
35 40 45 50 55 60

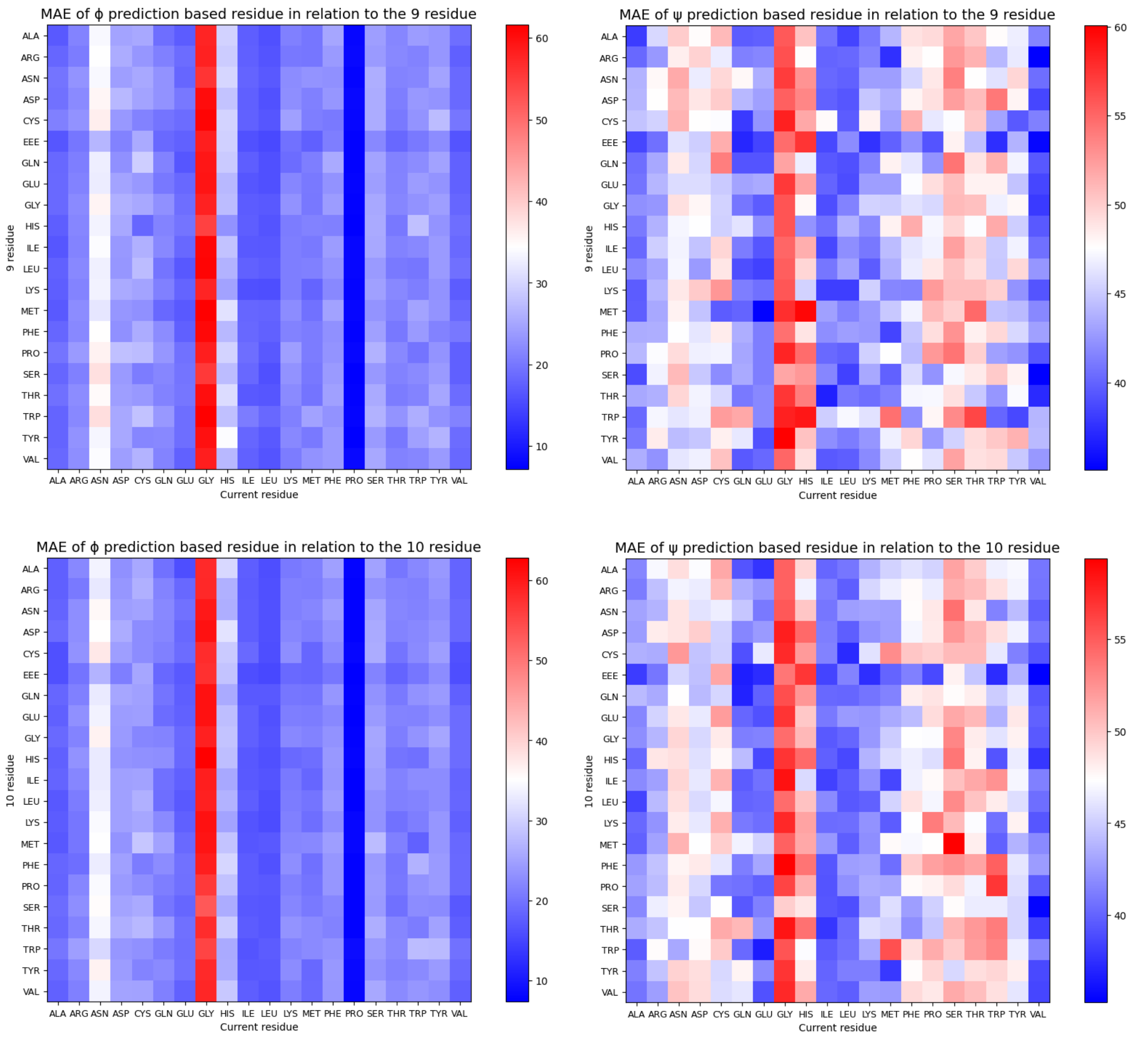

Figure S2. Mean absolute error of the current amino-acid residue in relation to the amino-acid residues at different positions of the sliding window for the ϕ (left) and ψ (right) dihedral angles.

Table S2. Real and predicted amino acid conformational propensites of the test dataset.

	Real			Predicted		
	**Alpha**	**Beta**	**Coil**	**Alpha**	**Beta**	**Coil**
**Ala**	4.48	2.97	0.90	4.71	2.75	0.90
**Arg**	2.21	2.02	0.65	2.25	1.96	0.67
**Asn**	1.44	1.93	0.98	1.05	2.11	1.18
**Asp**	2.15	2.51	1.18	1.98	2.28	1.57
**Cys**	0.40	0.78	0.17	0.35	0.85	0.16
**Gln**	1.72	1.44	0.50	1.70	1.40	0.56
**Glu**	3.45	2.14	0.85	3.60	1.95	0.89
**Gly**	1.34	1.54	4.70	1.20	1.21	5.17
**His**	0.78	1.14	0.39	0.68	1.17	0.46
**Ile**	2.24	3.09	0.30	2.02	3.40	0.20

**Leu**	4.24	3.80	0.97	4.24	3.77	1.00
**Lys**	2.65	2.24	0.81	2.66	2.16	0.88
**Met**	0.94	0.88	0.23	0.93	0.89	0.22
**Phe**	1.39	2.14	0.47	1.33	2.25	0.42
**Pro**	1.25	2.72	0.65	0.97	2.98	0.66
**Ser**	1.91	3.14	1.21	1.85	3.15	1.26
**Thr**	1.55	3.15	0.96	1.45	3.41	0.79
**Trp**	0.59	0.77	0.18	0.56	0.81	0.16
**Tyr**	1.21	1.95	0.44	1.09	2.10	0.41
**Val**	2.39	4.40	0.42	2.14	4.79	0.28

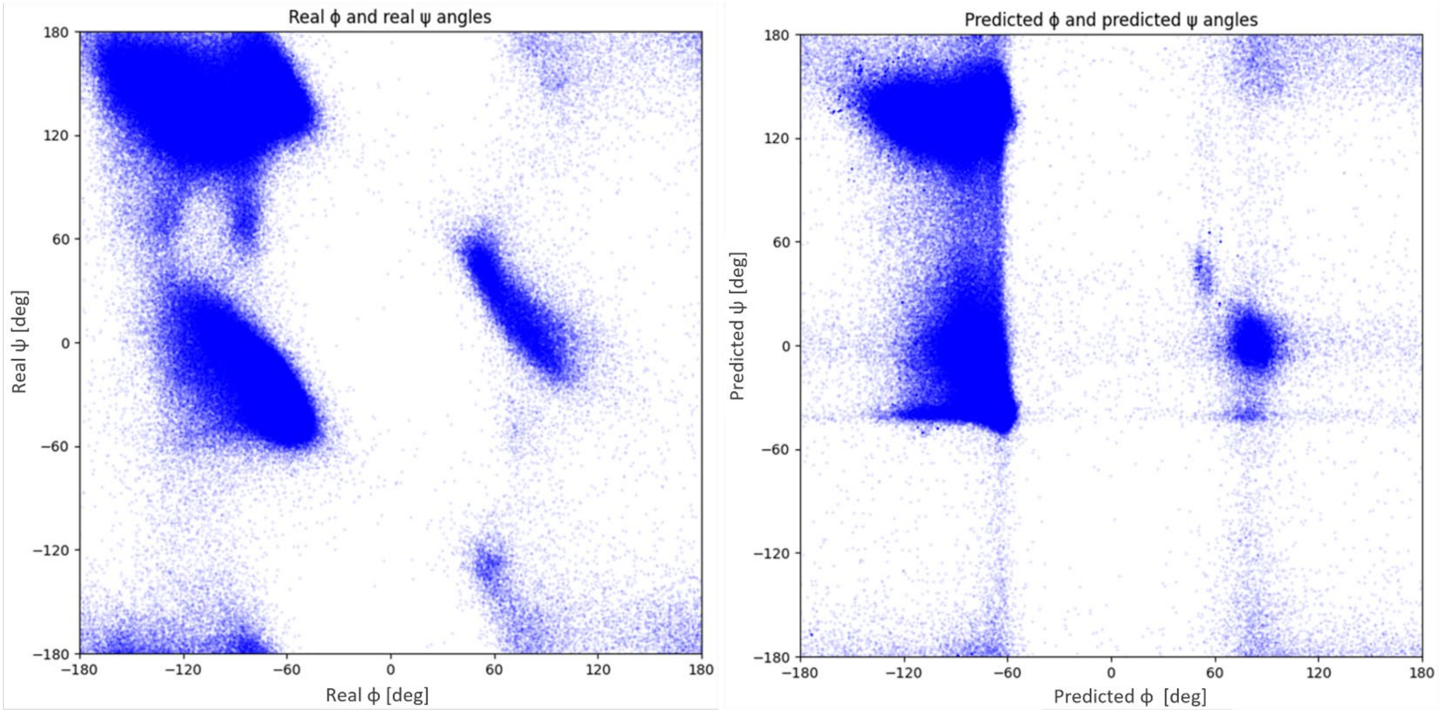

Figure 3. Ramachandran plots of actual and predicted dihedral angles.

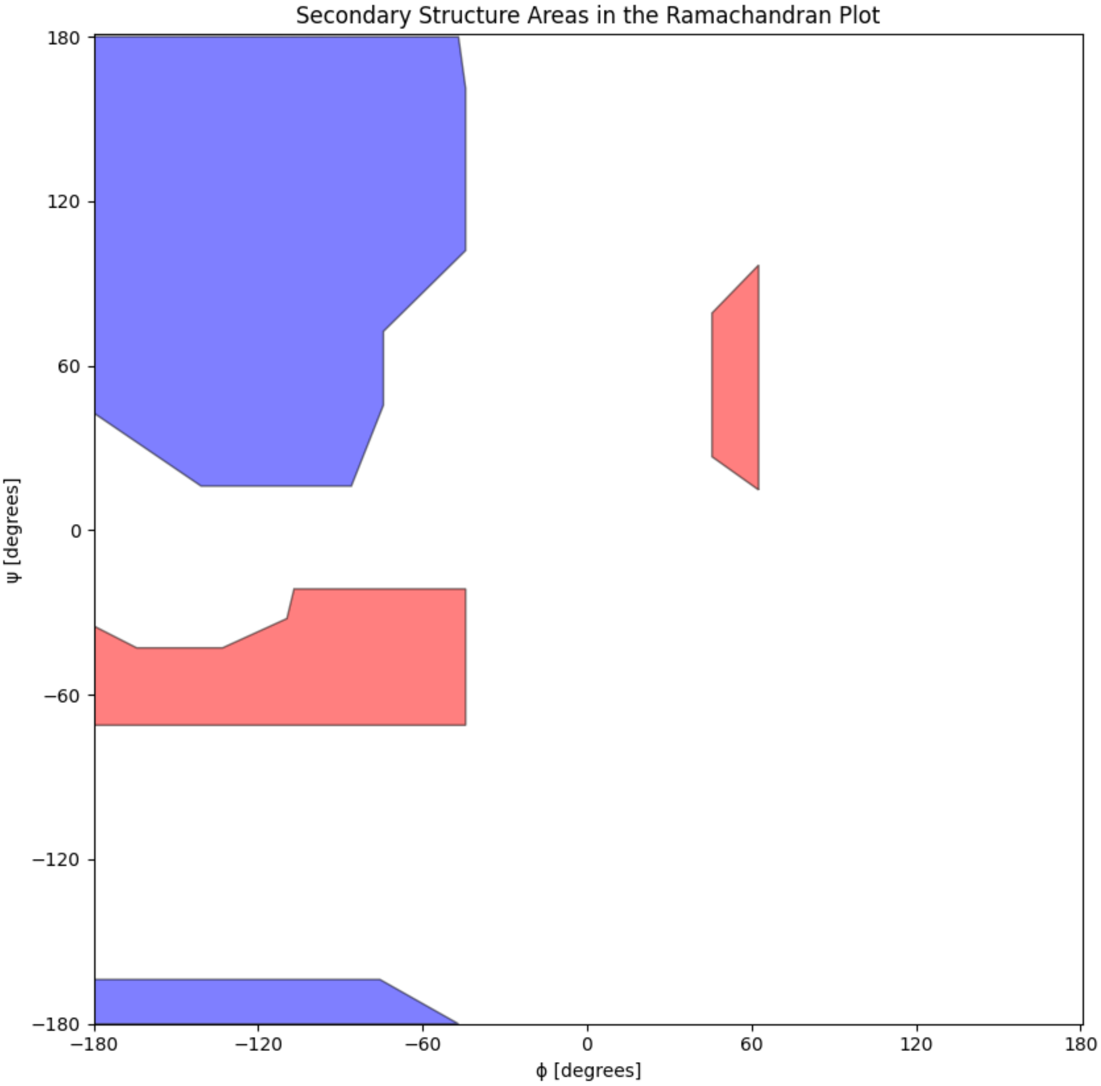

Figure S4. Secondary structure areas in the Ramachandran plot. The areas represent secondary structure elements: helices (red), sheets (purple), and undesignated (white). The areas are enclosed by the following points: helix = [(-180.0, -34.9), (-164.3, -42.9), (-133.0, -42.9), (-109.5, -32.2), (-106.9, -21.4), (-44.3, -21.4), (-44.3, -71.1), (-180.0, -71.1)] AND [(62.6, 14.7), (62.6, 96.7), (45.6, 79.2), (45.6, 26.8), (62.6, 14.7)]; sheet = [(-180.2, 42.9), (-140.8, 16.1), (-86.0, 16.1), (-74.3, 45.6), (-74.3, 72.5), (-44.3, 102.0), (-44.3, 161.1), (-46.9, 179.9), (-180, 180)] AND [(-180.0, -163.8), (-75.6, -163.8), (-46.9, -180.0), (-180.0, -180.0)].

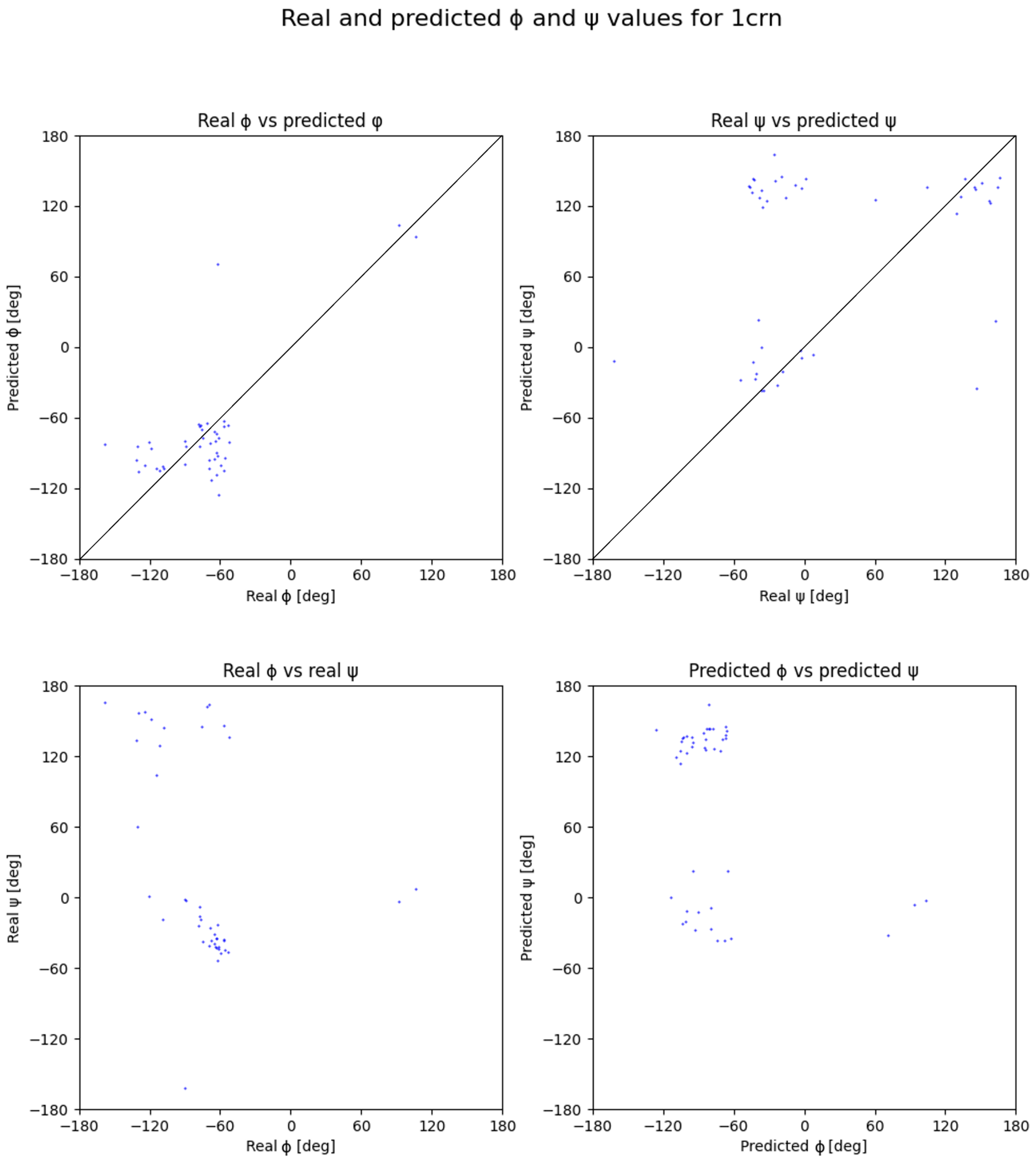

Figure S5. Real and predicted ϕ and ψ angles for the protein structure PDB ID: 1CRN (ϕ error = 24.91°; ψ error = 80.86°).

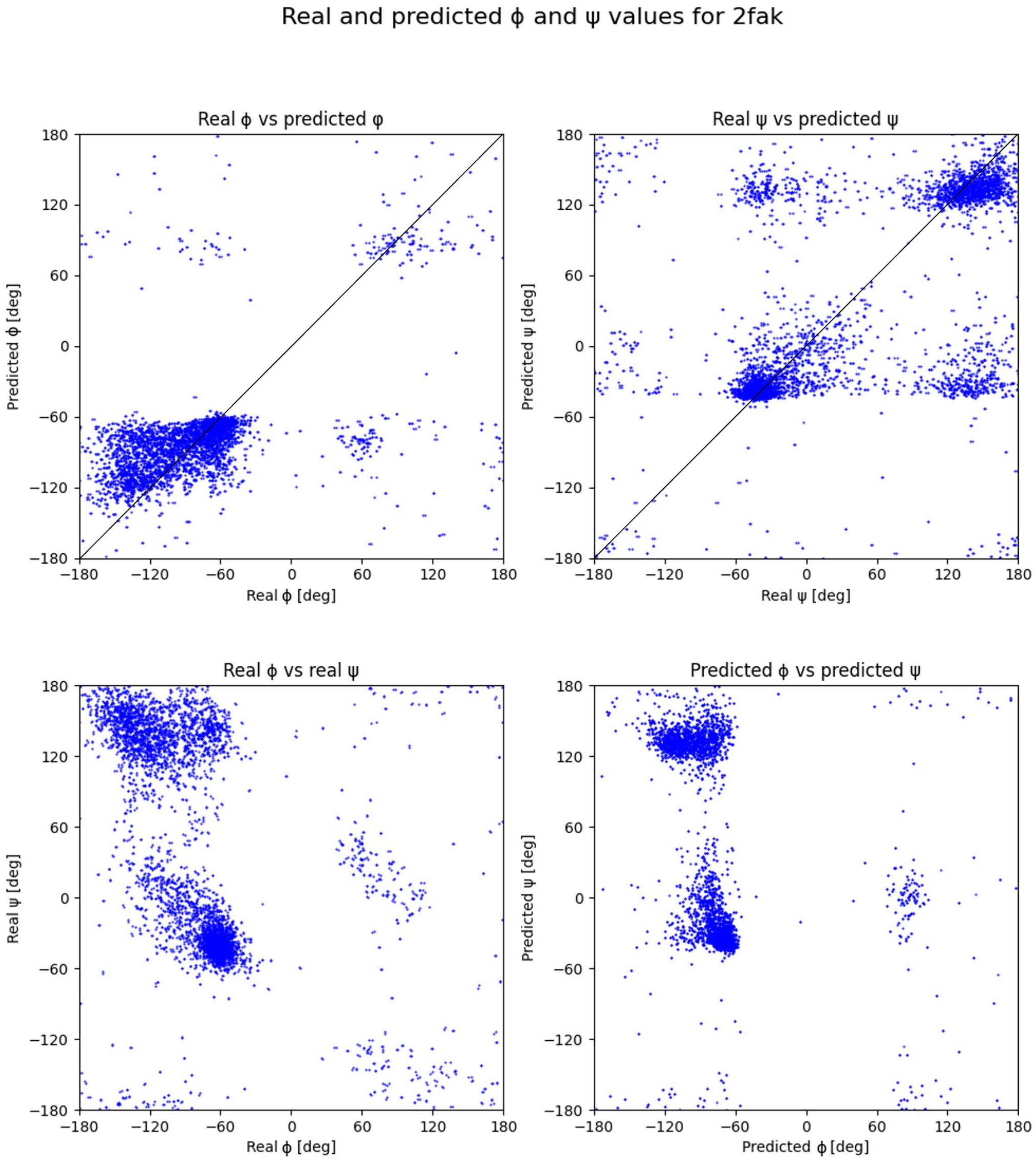

Figure S6. Real and predicted ϕ and ψ angles for the protein structure PDB ID: 2FAK (ϕ error = 25.71°; ψ error = 41.96°).

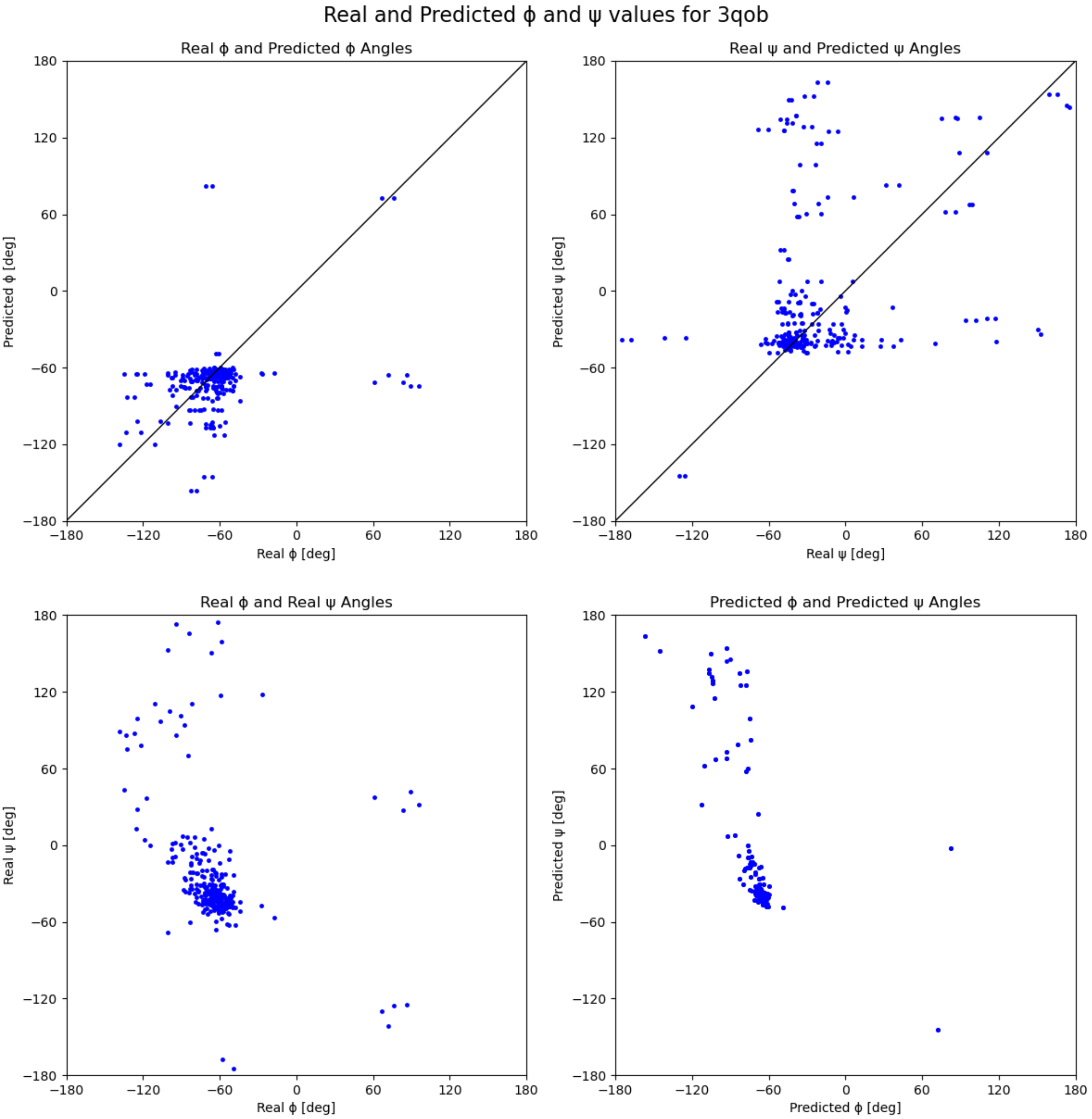

Figure S7. Real and predicted ϕ and ψ angles for the protein structure PDB ID: 3QOB (ϕ error = 17.76°; ψ error = 36.43°).

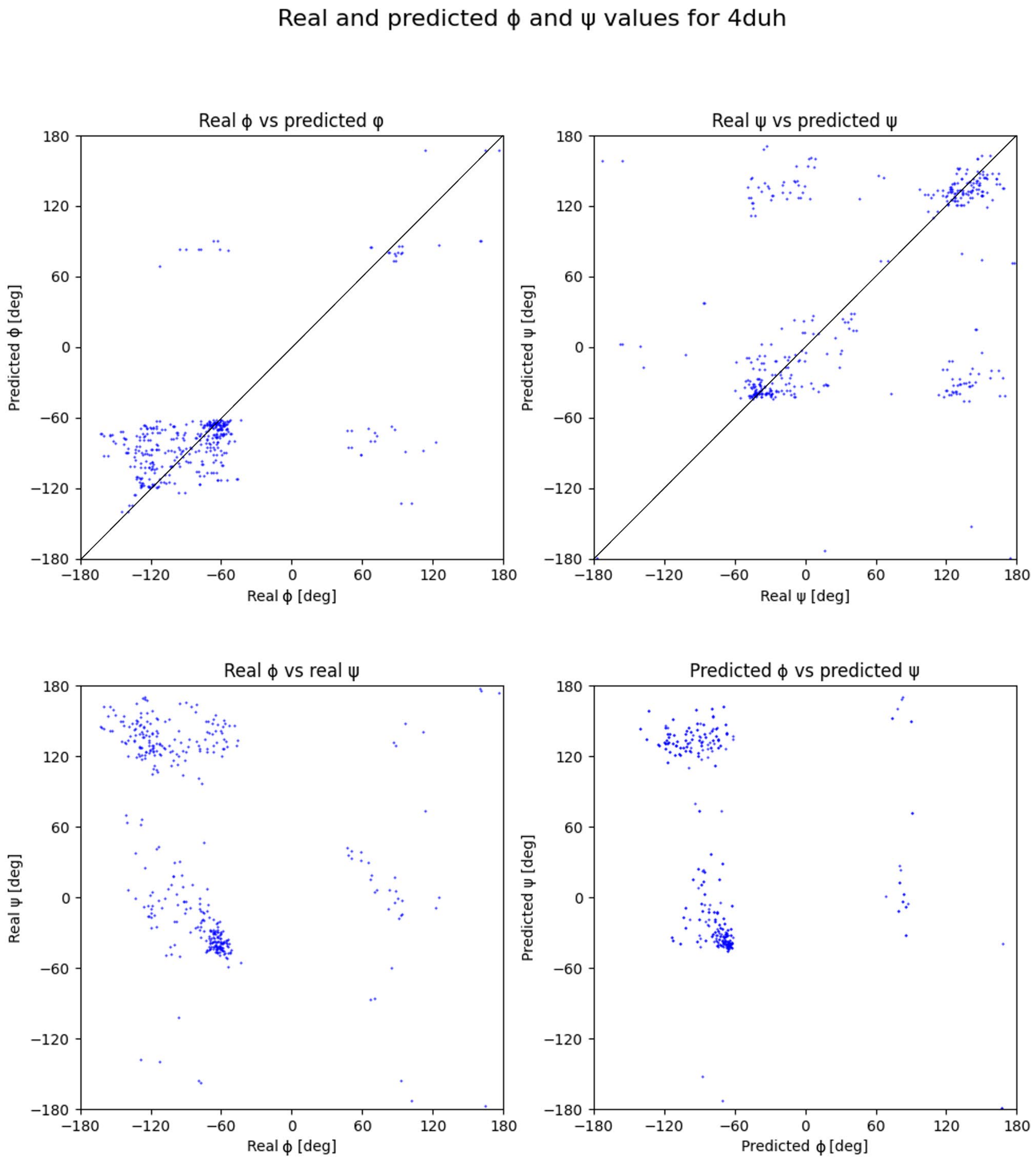

Figure S8. Real and predicted ϕ and ψ angles for the protein structure PDB ID: 4DUH (ϕ error = 29.86°; ψ error = 48.16°).

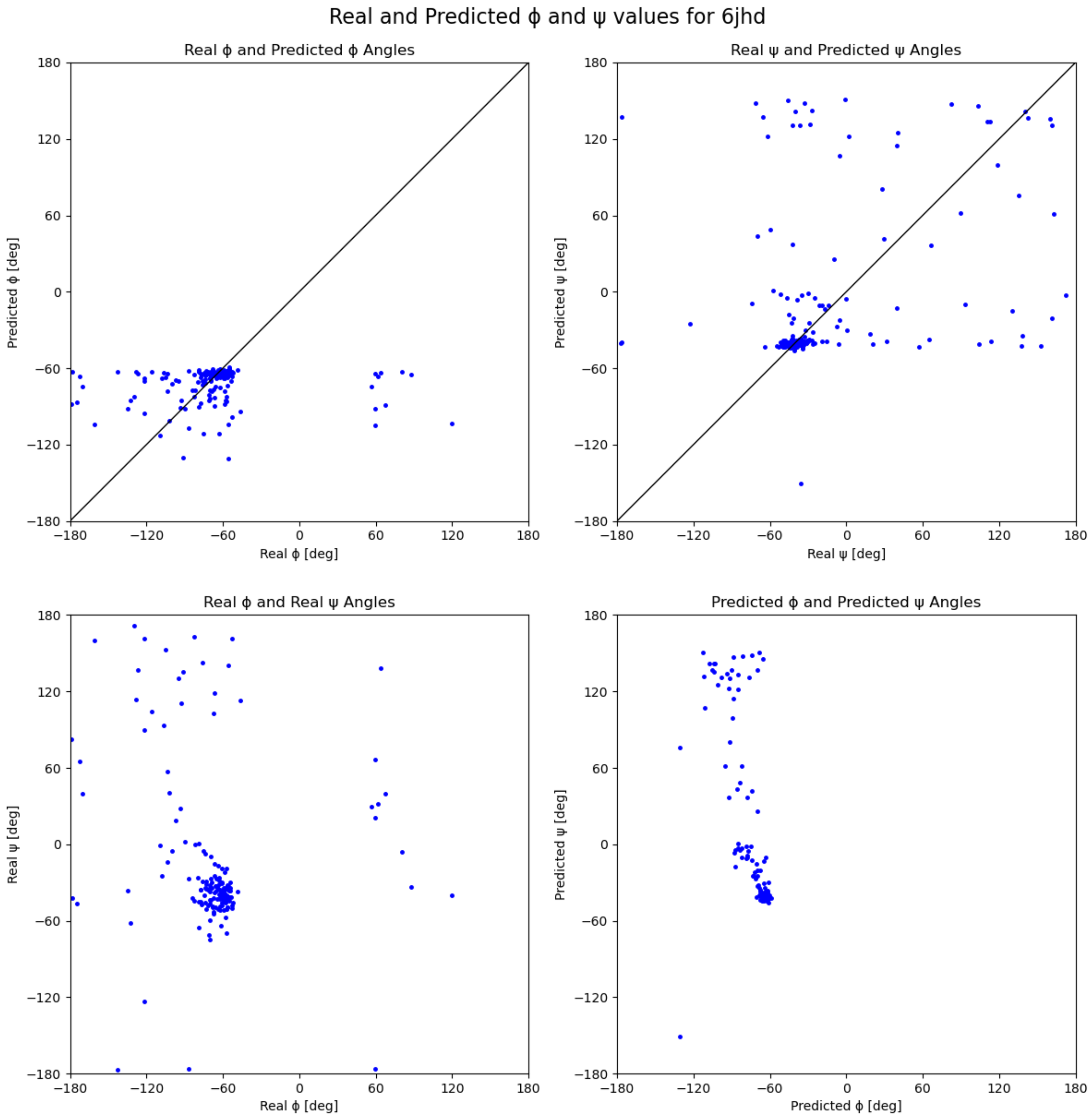

Figure S9. Real and predicted ϕ and ψ angles for the protein structure PDB ID: 6JHD (ϕ error = 24.57°; ψ error = 39.91°).

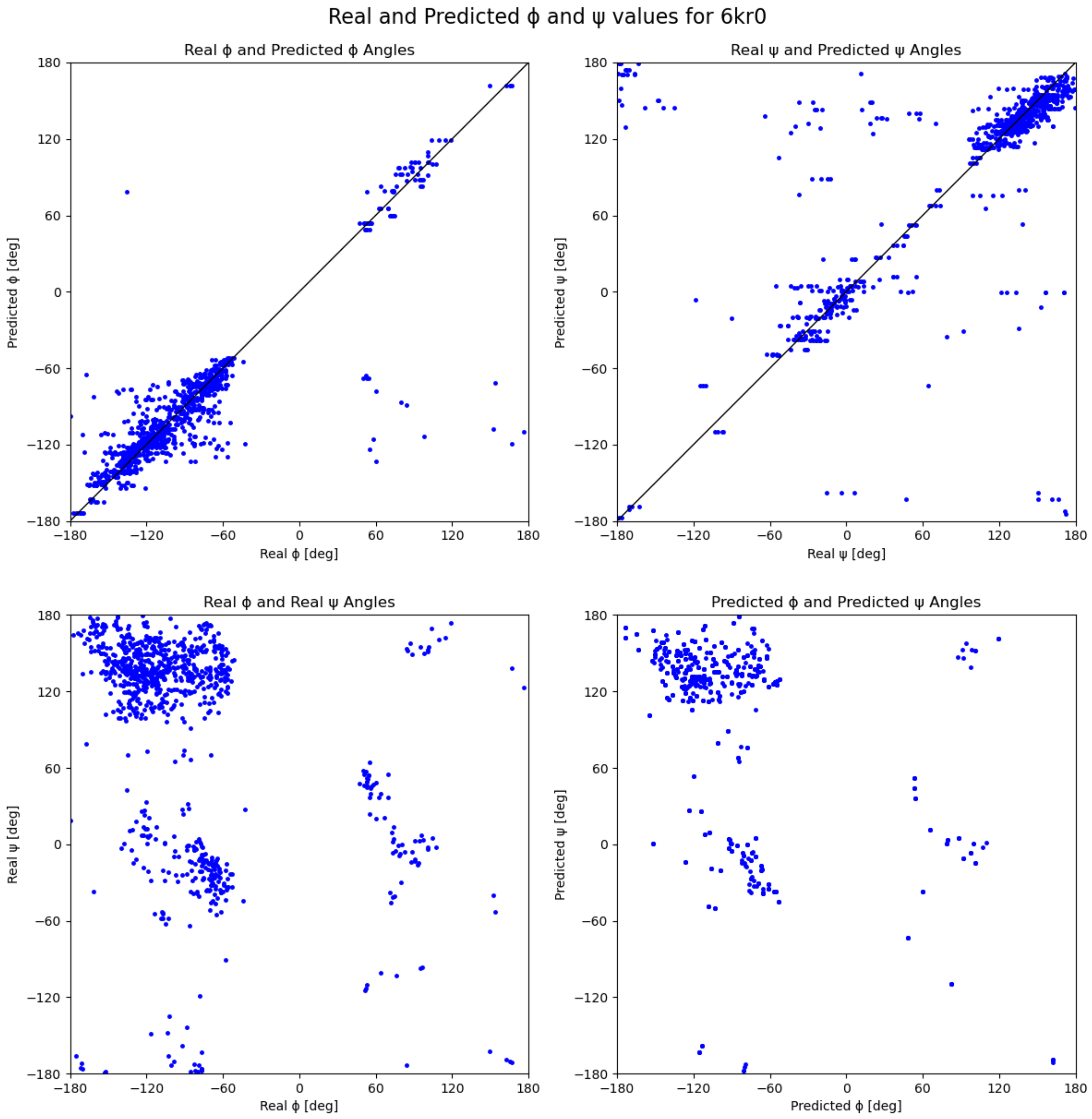

Figure S10. Real and predicted ϕ and ψ angles for the protein structure PDB ID: 6KR0 (ϕ error = 12.27°; ψ error = 16.10°).

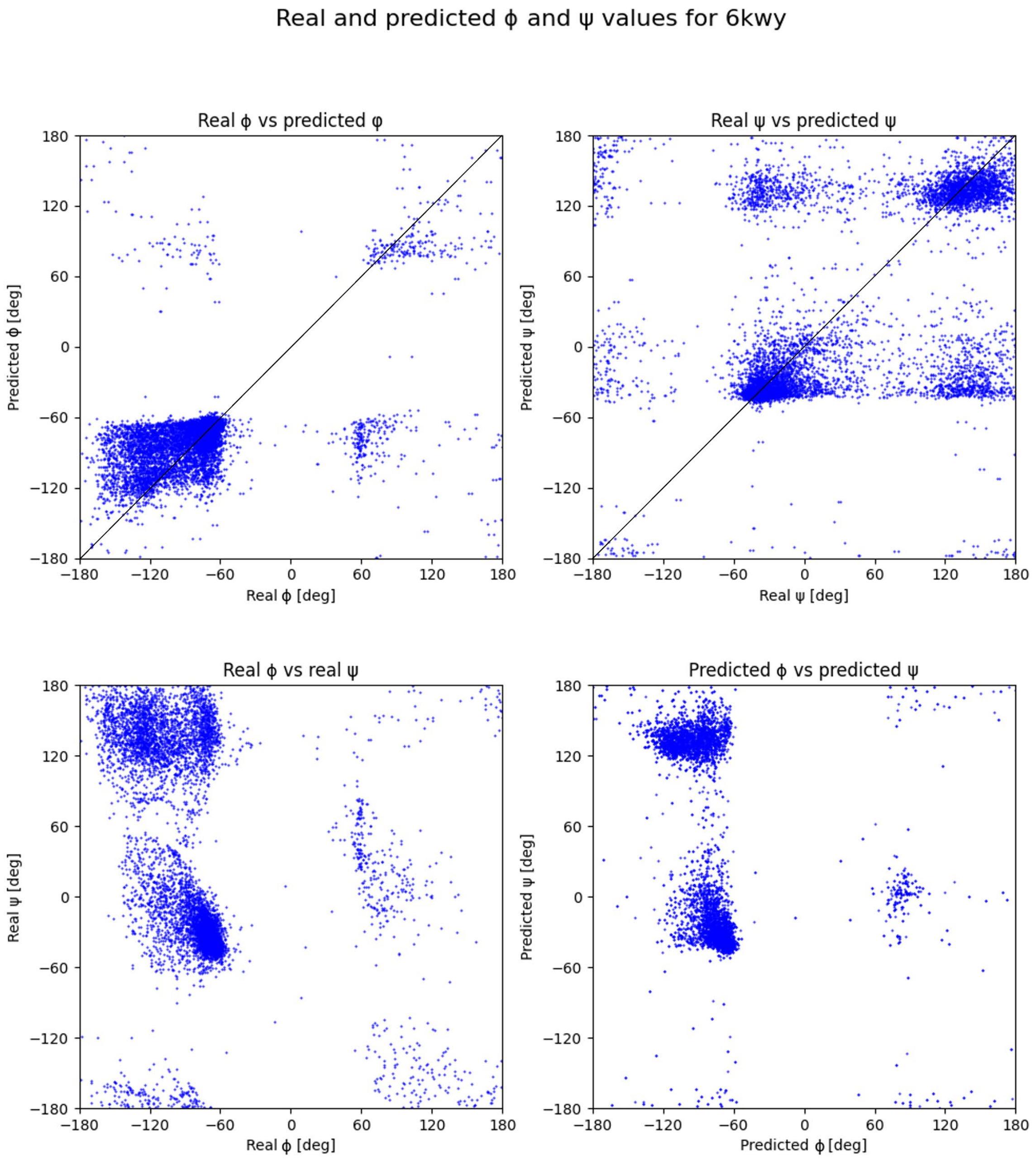

Figure S11. Real and predicted ϕ and ψ angles for the protein structure PDB ID: 6KWY (ϕ error = 22.78°; ψ error = 42.43°).

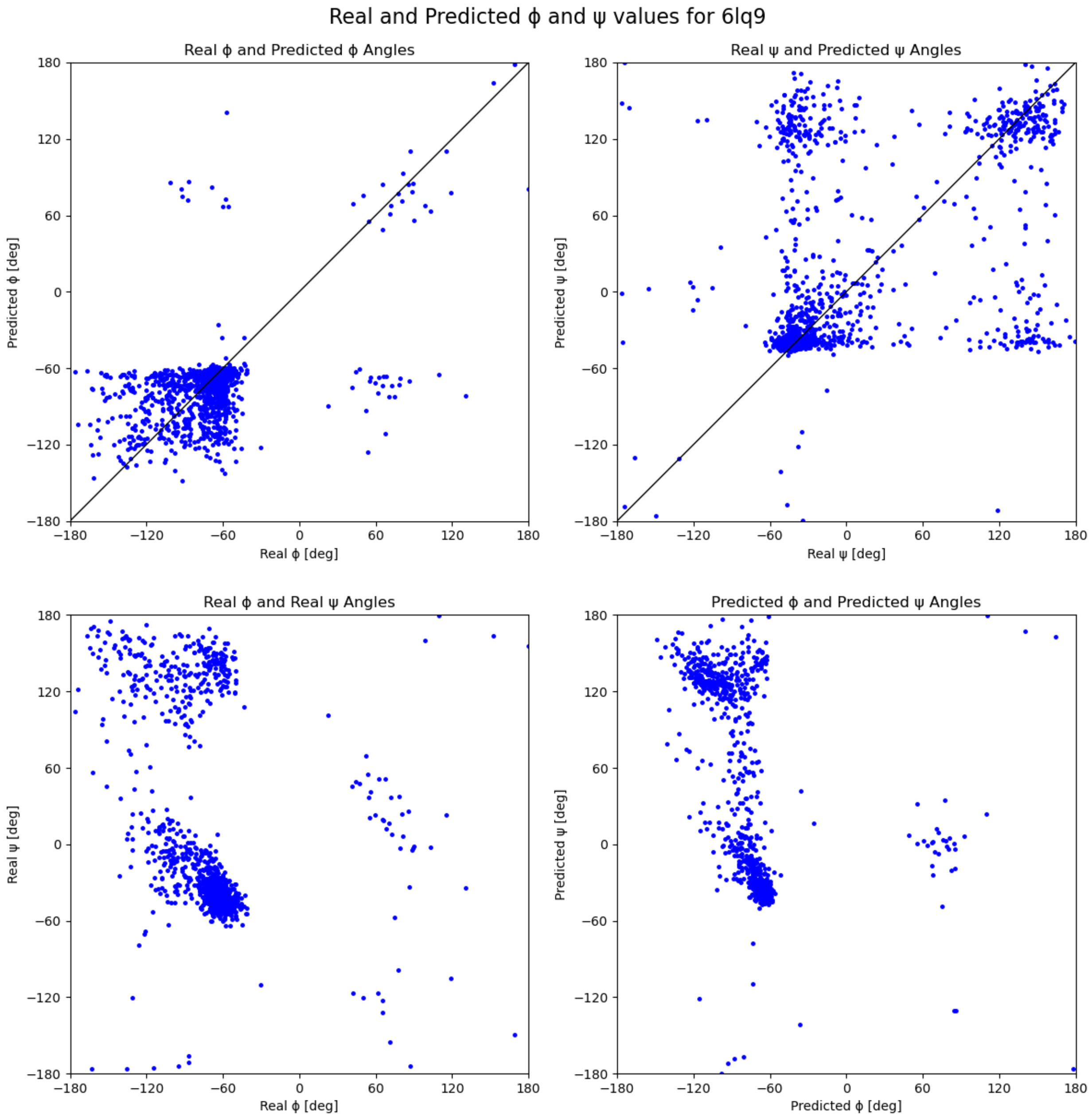

Figure S12. Real and predicted ϕ and ψ angles for the protein structure PDB ID: 6LQ9 (ϕ error = 18.83°; ψ error = 40.67°).

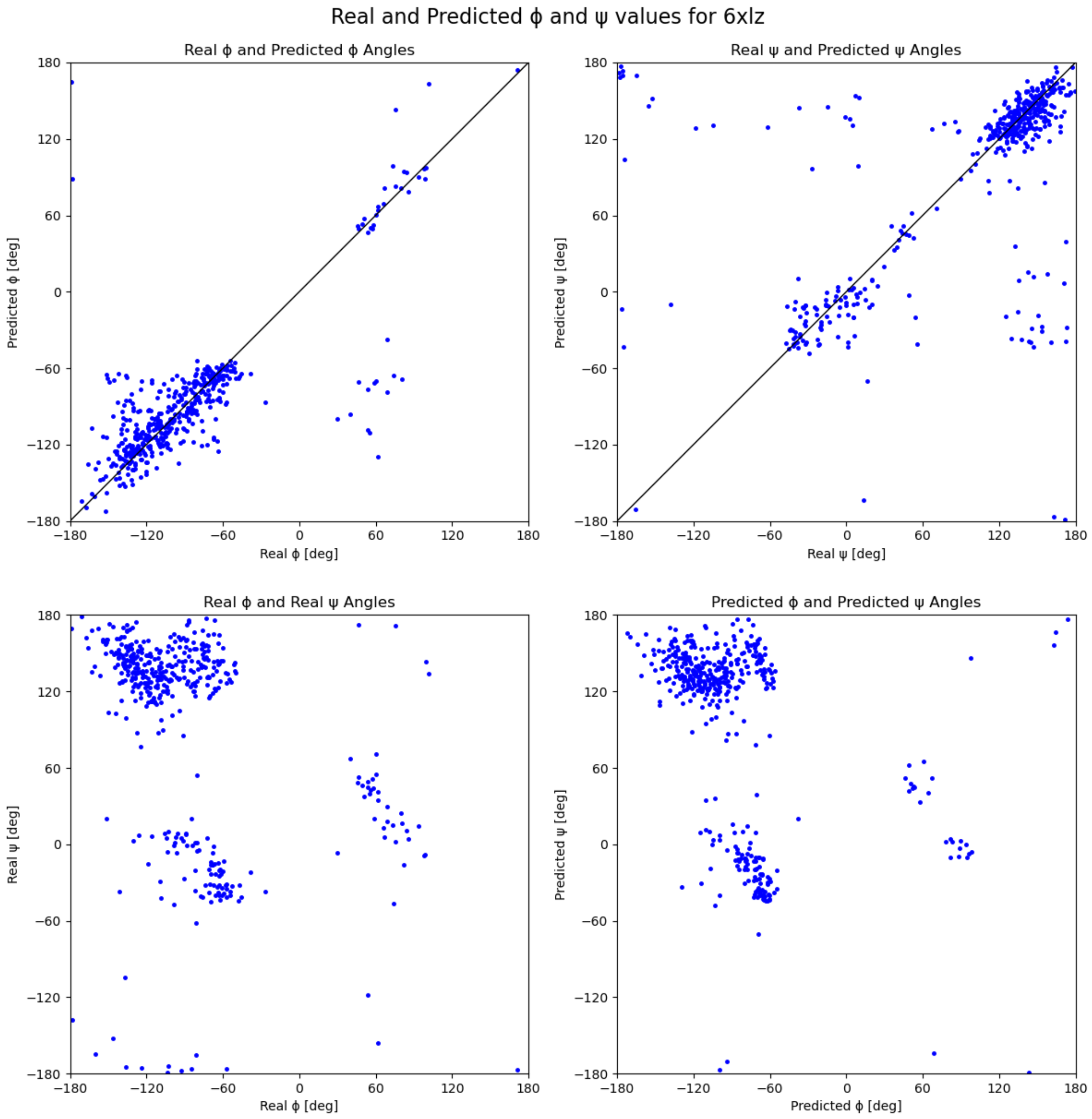

Figure S13. Real and predicted ϕ and ψ angles for the protein structure PDB ID: 6XLZ (ϕ error = 17.45°; ψ error = 23.29°).

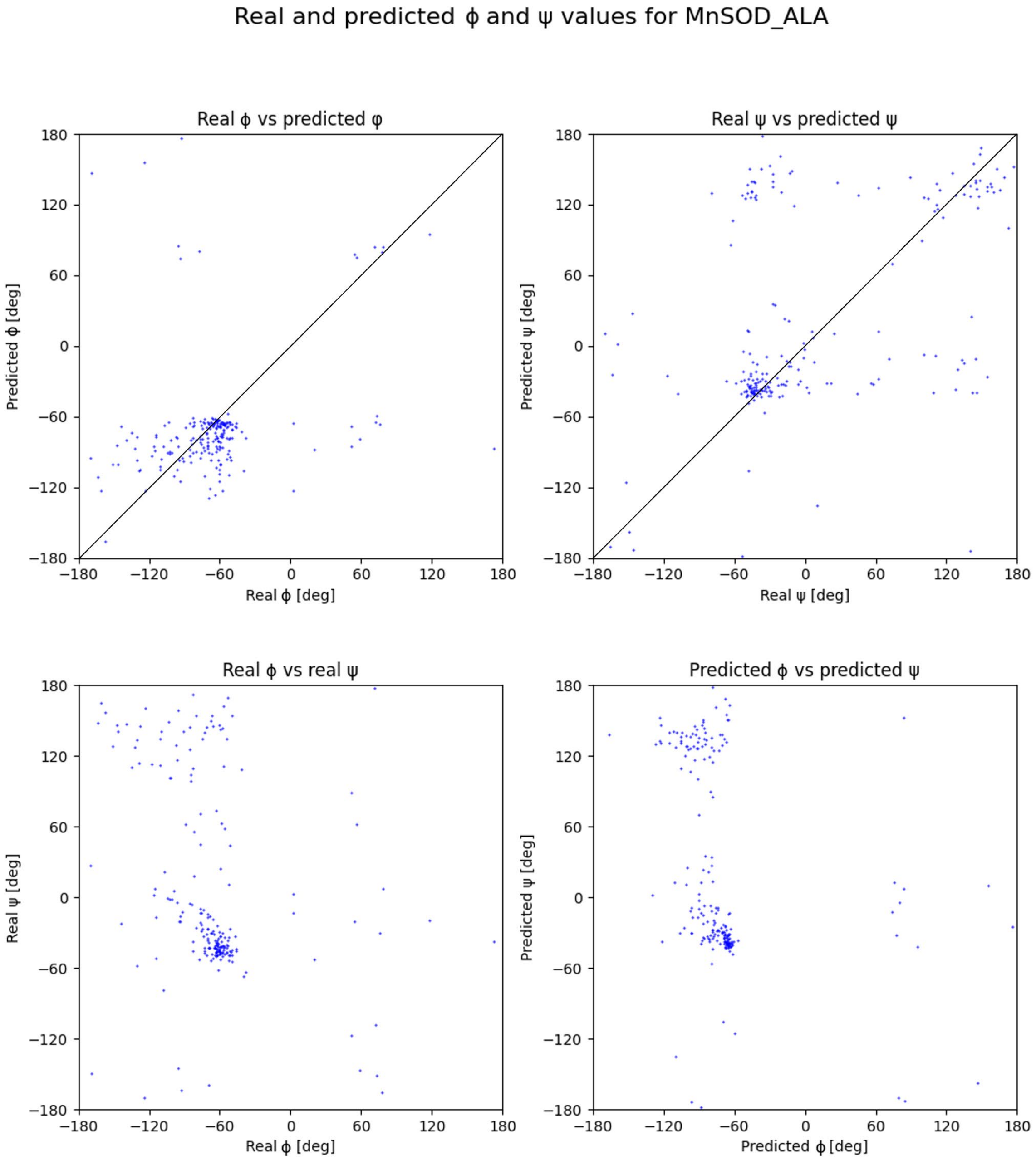

Figure S14. Real and predicted ϕ and ψ angles for the protein structure of MnSOD_ALA (ϕ error = 26.82°; ψ error = 51.12°).

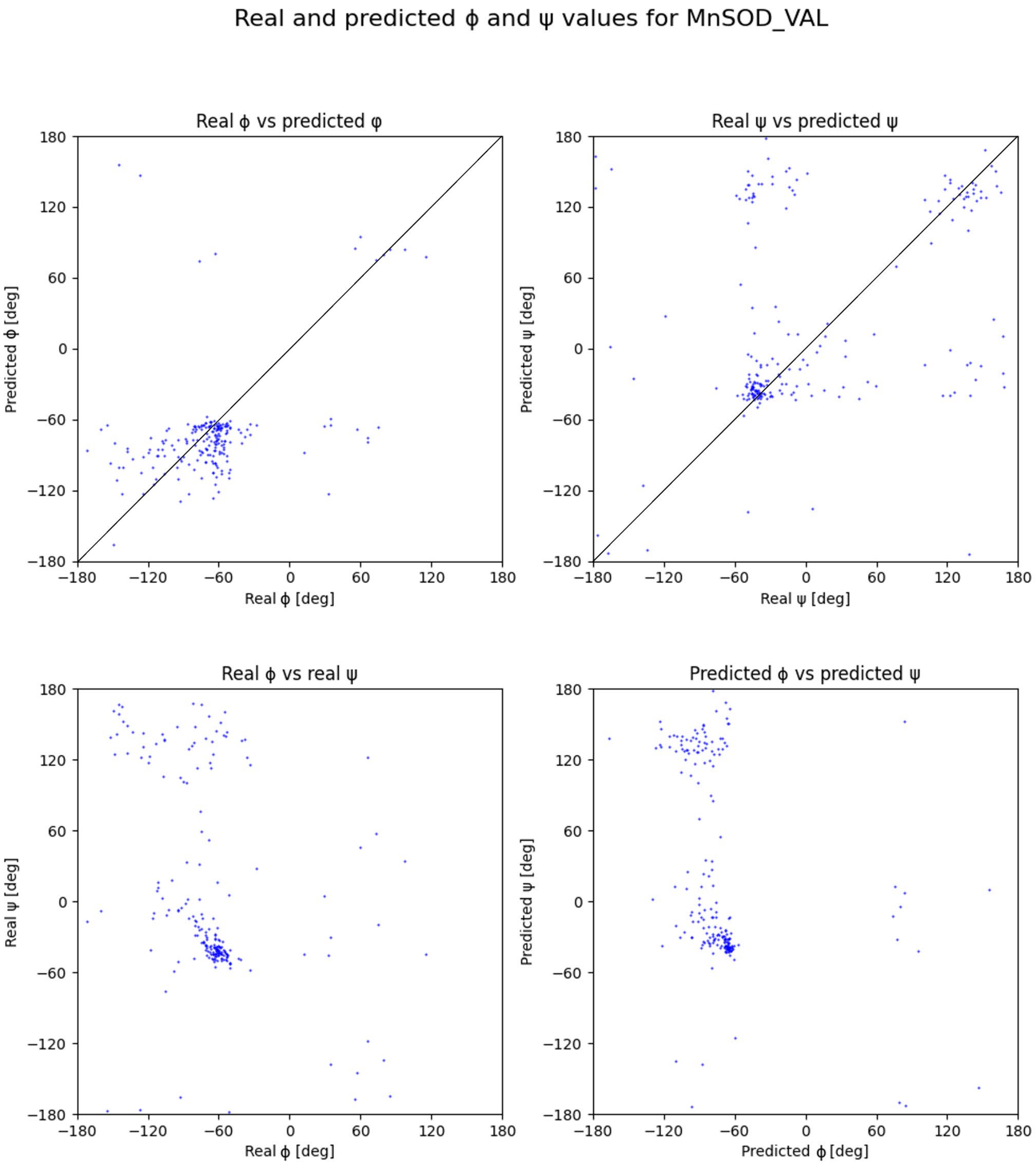

Figure S15. Real and predicted ϕ and ψ angles for the protein structure of MnSOD_VAL (ϕ error = 26.41°; ψ error = 50.44°).

Table S3. Per-model mean absolute errors of the predicted phi and psi dihedral angle of our model compared to an online tool DISSpred.

	**Our model**		**DISSpred**	
**PDB ID**	**Phi**	**Psi**	**Phi**	**Psi**
**1CRN**	22.63	77.65	32.70	49.86
**2FAK**	20.58	35.08	29.85	35.77
**4DUH**	28.65	54.18	42.60	85.49
**6KWY**	26.19	44.18	43.28	91.98
**6JHD**	24.57	39.90	25.76	34.35
**6KR0**	12.24	16.70	47.25	90.02
**6LQ9**	26.57	48.82	37.57	73.66
**3QOB**	18.12	37.80	19.94	32.03

**6XLZ**	16.59	27.03	43.40	81.24
**MnSOD-ALA**	26.69	50.94	31.06	60.28
**MnSOD-VAL**	27.65	50.16	31.99	58.17
**Average**	**22.77**	**43.86**	**35.03**	**62.99**

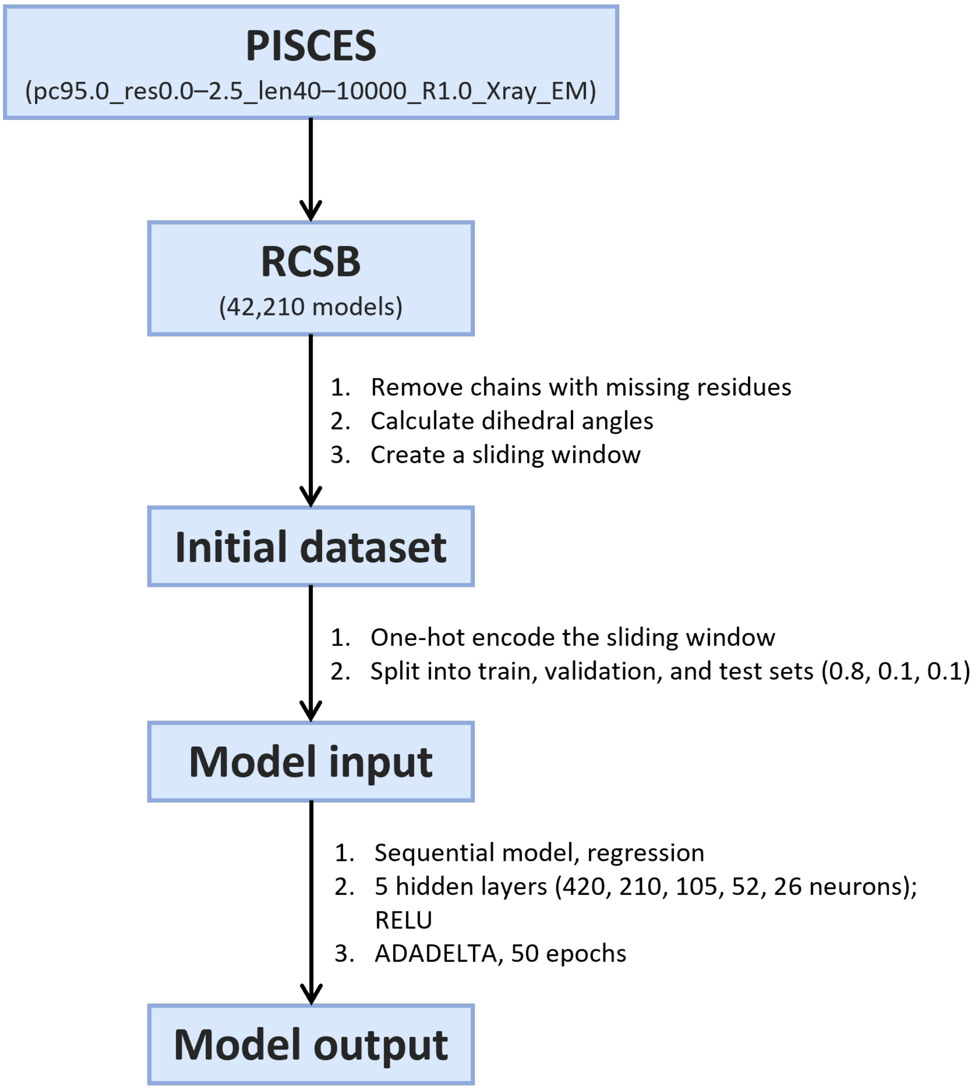

Figure S16. The process of generating output, which involves accessing the PISCES and RCSB datasets, converting the coordinates to dihedral angles, and transforming the sequence into a sliding window format.

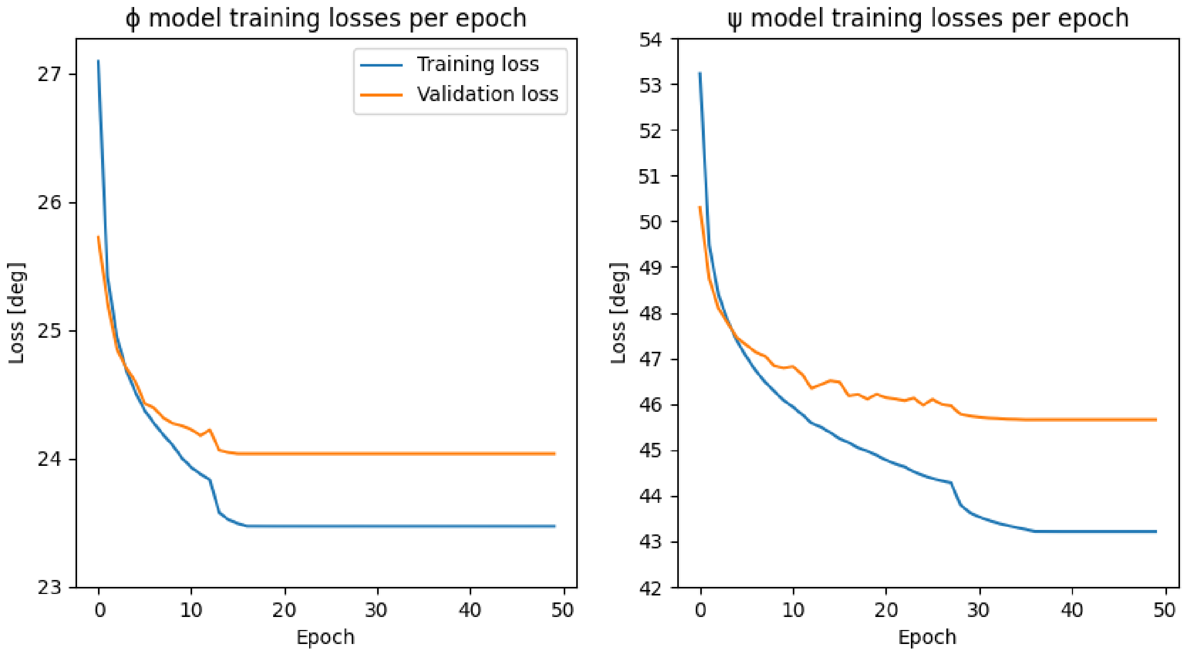

Figure S17. Training and validation loss function values during the ϕ and ψ model training.
